# Capability and accuracy of usual statistical analyses in a real-world setting using a federated approach

**DOI:** 10.1371/journal.pone.0312697

**Published:** 2024-11-14

**Authors:** Romain Jégou, Camille Bachot, Charles Monteil, Eric Boernert, Jacek Chmiel, Mathieu Boucher, David Pau

**Affiliations:** 1 Keyrus Life Science, Nantes, France; 2 Roche Medical Data Center, Boulogne-Billancourt, France; 3 Roche Informatics, Boulogne-Billancourt, France; 4 Roche Federated Open Science Solution, Basel, Switzerland; 5 Avenga, Warsaw, Poland; Huazhong University of Science and Technology, CHINA

## Abstract

**Methods:**

The objective of this project was to determine the capability of a federated analysis approach using DataSHIELD to maintain the level of results of a classical centralized analysis in a real-world setting. This research was carried out on an anonymous synthetic longitudinal real-world oncology cohort randomly splitted in three local databases, mimicking three healthcare organizations, stored in a federated data platform integrating DataSHIELD. No individual data transfer, statistics were calculated simultaneously but in parallel within each healthcare organization and only summary statistics (aggregates) were provided back to the federated data analyst.

Descriptive statistics, survival analysis, regression models and correlation were first performed on the centralized approach and then reproduced on the federated approach. The results were then compared between the two approaches.

**Results:**

The cohort was splitted in three samples (N1 = 157 patients, N2 = 94 and N3 = 64), 11 derived variables and four types of analyses were generated. All analyses were successfully reproduced using DataSHIELD, except for one descriptive variable due to data disclosure limitation in the federated environment, showing the good capability of DataSHIELD. For descriptive statistics, exactly equivalent results were found for the federated and centralized approaches, except some differences for position measures. Estimates of univariate regression models were similar, with a loss of accuracy observed for multivariate models due to source database variability.

**Conclusion:**

Our project showed a practical implementation and use case of a real-world federated approach using DataSHIELD. The capability and accuracy of common data manipulation and analysis were satisfying, and the flexibility of the tool enabled the production of a variety of analyses while preserving the privacy of individual data. The DataSHIELD forum was also a practical source of information and support. In order to find the right balance between privacy and accuracy of the analysis, set-up of privacy requirements should be established prior to the start of the analysis, as well as a data quality review of the participating healthcare organization.

## Introduction

**Fig 1:**
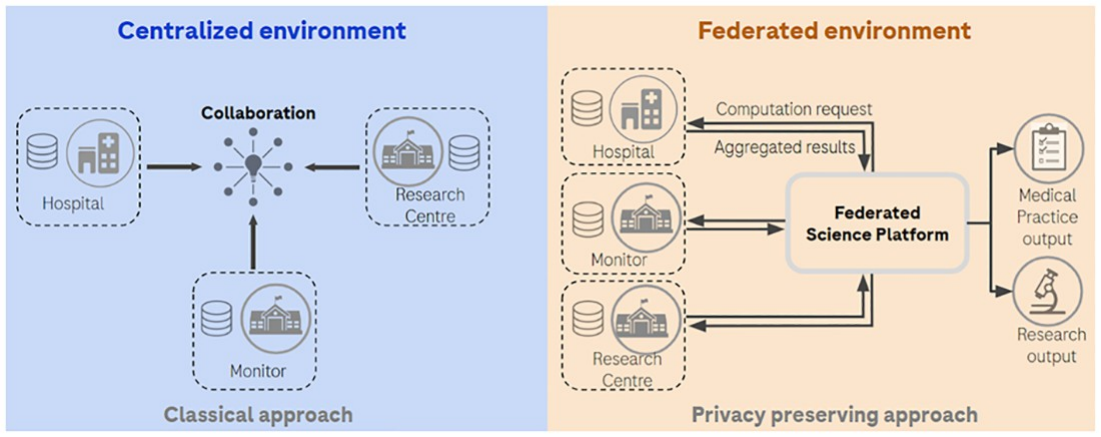
Overview of the centralized and federated environments.

In the classical approach of clinical trials, data from healthcare providers is transferred and stored in a centralized environment for statistical analysis (Fig 1). Due to legal, ethical or informed consent restrictions that protect privacy of the patient, individual-level data cannot always be easily and timely shared between organizations in a central environment [1]. Due to these increasing regulations on data sharing to protect sensitive data, such as the General Data Protection Regulation (GDPR) in Europe [2] and similar regulations in other regions [3], the healthcare organizations tend to retain ownership of their data in the context of collaboration, keeping control on access to data and its value.

Data sharing and collaboration have been significantly improved with new technologies, and the Federated Analysis (FA) platform is one of the solutions to decentralize data [4]. FA enables the generation of statistical analyses without data transfer agreements between healthcare organizations. Individual-level data remains under the control of the provider. Only computational queries and corresponding aggregated results are transferred in an FA environment, which is respectful of data privacy and property of each data source, in accordance with regulations and informed consent requirements (Fig 1).

The area of FA is new and developing rapidly, there is a lot of research, and more and more are ready to use patterns and tools. In the landscape of existing FA tools, by its maturity and the strength of its community, data aggregation through anonymous summary-statistics from harmonized individual-level databases (DataSHIELD) [5] is recognized as a potential solution for privacy preserving analysis.

This project aims to provide learnings and insights for the future use of DataSHIELD by assessing its capability and accuracy of common statistical analyses in a real-world setting compared to the classical centralized approach. The practical use of DataSHIELD within the privacy preserving federated environment will allow this evaluation. The comparison of the results generated through DataSHIELD with those generated through the centralized approach will support a preliminary review of the accuracy of the results. The objective of this project was to determine the capability of an FA approach using DataSHIELD to maintain the level of results of a classical centralized analysis in a real-world setting.

## Material

DataSHIELD is a software framework for secure bioscience collaboration that enables statistical analysis of individual-level data from multiple healthcare providers without transfer of data. DataSHIELD takes the analysis to the data by sending analysis requests from a central machine to multiple data-holding machines storing the harmonized data to be co-analysed simultaneously. The individual-level data remains within each healthcare provider and only non-disclosive summary data is shared with the data analyst.

DataSHIELD is an R-based programming tool that provides functions to assist the statistical analyses from data preparation to analysis [6]:

- Data preparation functions: creation or modification of variables needed for the analysis (coercing, variable manipulation or creation).
- Administrative functions: support and information setting functions.
- Data analysis functions: creation of statistical output and generalized linear modeling (data structure queries, summary statistics, matrices, tables, survival analysis, distribution generating, modeling).
- Data presentation functions: creation of graphical output.

In case the base functionality package does not cover specific data derivation or analysis needs at the time of the analysis, DataSHIELD offers the flexibility to define custom functions or to use and update existing custom functions provided by the DataSHIELD community [7]. However, community packages are supplied without warranty, and it is the responsibility of the user to ensure that sufficient data disclosure controls are implemented by the function.

## Project data and ethic statement

This research was based on data from a French real-world retrospective study collected from patient’s electronic medical records. The raw data represented a non-interventional study describing the epidemiology and the therapeutic management of 315 patients treated for early breast cancer among 57 sites in France. The start date of data extraction was May 2019, and the end date of data extraction was September 2019. Based on French regulations, this study belonged to the category of healthcare research involving secondary use and analysis of data. This retrospective study complied with the French “Commission nationale de l’informatique et des libertés–CNIL” (translated to National Commission on Informatics and Liberty), reference methodology 004 (MR-004). The cohort study was approved by the Institutional Review Board/Independent Ethics Committee on May 03, 2018, prior study set-up. All the patients received written information before any trial-related activities were carried out. In addition, the analyses of this research were performed on an anonymised synthetic version of the data, no author had access to information that could identify individual participants during or after data collection.

## Project infrastructure set-up

The raw database is composed of seven different datasets: demographic characteristics, disease diagnostic, surgery, pathological complete response (pCR), adjuvant treatments, patients follow-up and patients lost to follow-up information.

**Fig 2:**
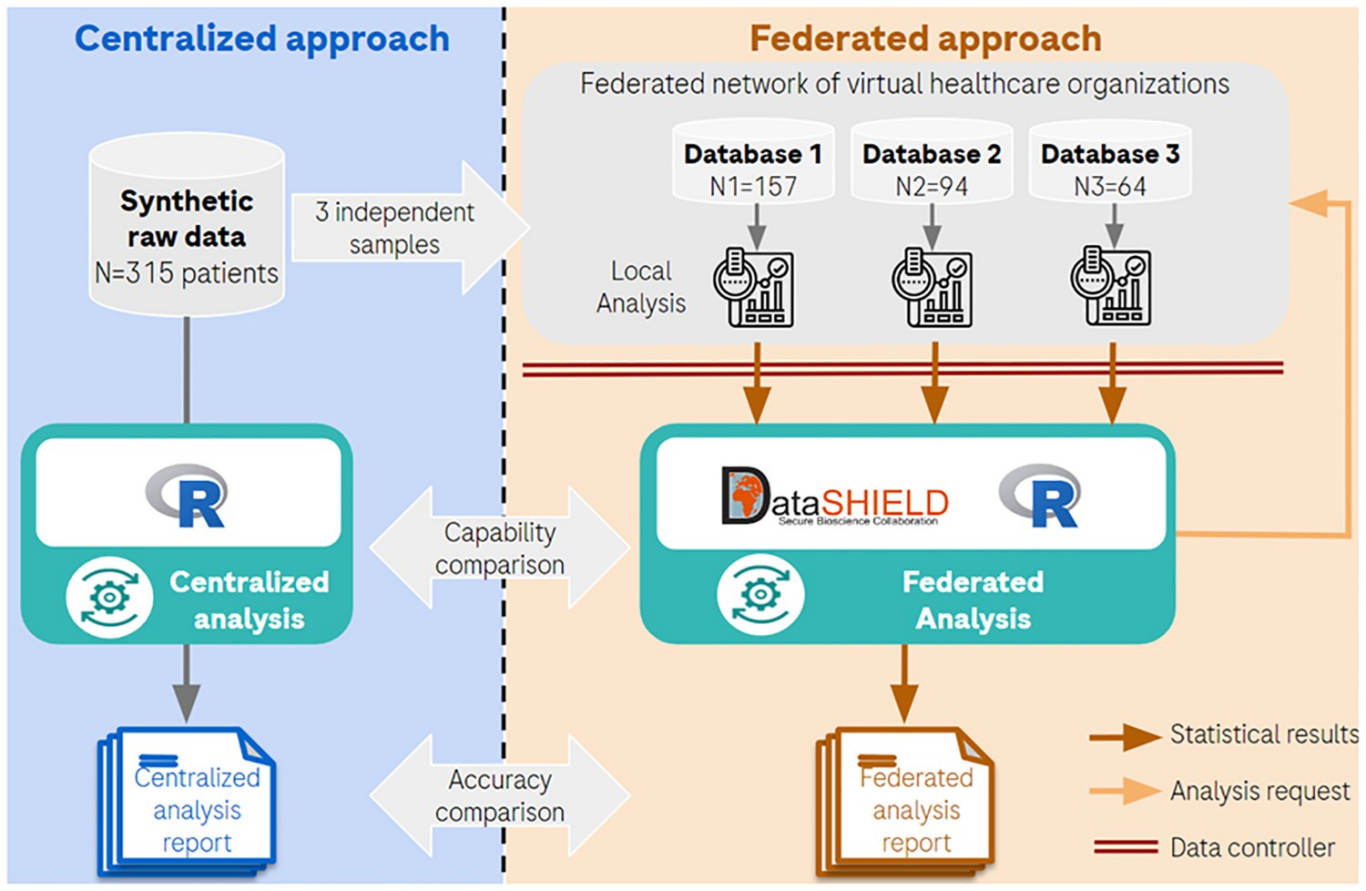
Overview of the implementation of the project environment.

Based on this synthetic raw database, a virtual federated network of three healthcare organizations was generated by randomly splitting the data into three distinct local databases. Each database represented a healthcare organization of different size (N1 = 157 patients, N2 = 94 and N3 = 64, respectively). This splitting process ensured that each database contained a unique part of the patient by maintaining a similar data structure. These three databases were then stored on a data platform integrating DataSHIELD functionality (Fig 2). This environment is a practical implementation of an FA setup in a multi-organization context while preserving data privacy (no individual data transfer and storage).

In order to assess the existing capabilities of DataSHIELD, the programming intent of this project was to use built-in distributed R functions from community packages, available at the start of the project in 2022, as a priority over writing custom functions. The privacy guardrails, including disclosive control filters [8], were set at the default level in each local data repository server. These filters are key in the DataSHIELD infrastructure to protect privacy and ensure that sensitive data is not disclosed during statistical analysis. R version 4.2.2 and DataSHIELD version 6.2 were used.

## Statistical methods

11 derivations were performed on both the centralized and federated data to facilitate further statistical outputs and modeling. The following data transformations were performed:

Class factors:

- Age group in years (<40, [40–49], [50–59], [60–69], > = 70).
- BMI group in kg/m2 (<25, [25–29], > = 30).
- T class et N class.

Duration:

- Follow-up duration (in years).
- Time from diagnostic to surgery (in months).
- Time from diagnostic to progression (in years).
- Age at adjuvant treatment initiation of Herceptin (in years).
- Treatment duration (in months).
- Time from surgery to adjuvant treatment initiation of Herceptin (in days).
- Time to event/censor for survival analysis (in days).

Binary outcomes (multilevel conditions):

- pCR results (Yes, No).
- Indicator of event/censored for survival analysis.

We conducted the following types of analysis:

- **Descriptive** statistics with mean, standard deviation (SD), median, quantiles for continuous variables and proportions for categorical variables.
- **Survival** analysis [9] to visualize and identify predictive factors for progression-free survival (PFS).
- **Logistic** regression [10] to identify predictive factors for pCR.
- **Correlation** matrix to assess associations between variables.

The analyses were first generated in the centralized environment using R programming, and then reproduced in the privacy preserving federated environment using R-based DataSHIELD programming (Fig 2).

DataSHIELD was evaluated by assessing the capability and accuracy of the reproduced statistical analyses compared to those derived from the centralized approach. The reproducibility was assessed by evaluating which analyses could be produced using DataSHIELD. The accuracy of the results was assessed by comparison with those obtained using the centralized approach. The following evaluation were performed:

- Descriptive statistics included evaluation of difference in proportion for categorical outcome and difference in mean/SD/median for continuous outcome. The selected rules for determining similarity were defined as an absolute difference of 5% or less.
- Survival analysis included checking the survival probabilities from the K-M method, hazard ratio (HR) and global effect p-value from the Cox proportional hazards model.
- Logistic analysis included checking the odds ratio (OR) and global effect p-value from logistic regression.
- In the correlation matrix, the p-value obtained by Chi-square/Fisher’s exact test or ANOVA global effect p-value was checked.

## Results

### Data transformation

Derivation of class variables from existing numeric variables (such as BMI or age groups) and derivation of duration between two existing date variables (such as age at diagnosis) were successfully performed in the FA environment, using dssDeriveColumn [11], ds.Boole and ds.assign functions.

Survival analysis involves selecting the appropriate event and censoring dates to perform the analysis. The reference start date was defined as the adjuvant initiation start date, the event date was the date of progression or death, and the censoring date for patients without event was the last on-treatment adjuvant date. Due to data disclosure restrictions in DataSHIELD, the selection of event/censor date was considered as sensitive information for patient privacy, and therefore the use of the built-in DataSHIELD function for such derivation was not possible. In order to perform the required derivation, the solution was to develop a specific custom function to allow data selection on the server side. The same situation arose for more complex multi-level derivations, where custom functions were also created.

In addition, subgroup flags had to be created for the selection of patients having received a specific medication. These selections were possible in FA, using the ds.dataFrameSubset function. However, data disclosure restrictions limited the reporting of subgroups with fewer than three patients.

The use of a combination of DataSHIELD base functions and analysis-specific custom functions allowed the derivation of all variables needed to meet the analysis requirements (see functions in Appendix 1). This outcome shows the capability and flexibility of DataSHIELD to manipulate and transform data in an FA environment while maintaining data privacy.

### Descriptive statistics

Most of the analyses were descriptive, covering a total of 73 variables, 25 continuous and 48 categorical. In FA, descriptive statistics were calculated simultaneously but in parallel within each local database and then summary statistics were provided back to the federated analyst. For categorical parameters, the sums of all available observations in each source database (and the corresponding proportion) were calculated (dh.getStats [12] and ds.table functions). For continuous variables, the statistics were weighted by the number of patients with a non-missing value from each source database (ds.meanSdGp function), allowing proportional contribution of each database to the aggregated result (see aggregation methods in Appendix 2). All of these analyses were performed using the built-in DataSHIELD functions.

Table 1 shows the main characteristics of the data obtained from the centralized and federated approaches.

**Table 1:** Description of the data in the centralized and federated approaches.

Characteristic	Centralized N = 315	Federated* N = 315	Local source database		
			Database 1 N = 157	Database 2 N = 94	Database 3 N = 64
Age at adjuvant treatment (years)					
Mean (SD)	52.23 (11.82)	52.23 (11.82)	51.69 (11.36)	53.24 (12.59)	52.08 (11.84)
Median	52.0	52.1	52.0	52.0	52.5
Q1—Q3	43.0–60.5	43.3–60.7	44.5–59.0	43.0–64.0	41.0–60.0
Min—Max	29–77	NR—NR	NR—NR	NR—NR	NR—NR
Missing	12	12	6	4	2
<40	53 (17.5%)	53 (17.5%)	27 (17.9%)	15 (16.7%)	11 (17.7%)
[40–49]	75 (24.8%)	75 (24.8%)	37 (24.5%)	24 (26.7%)	14 (22.6%)
[50–59]	91 (30.0%)	91 (30.0%)	52 (34.4%)	19 (21.1%)	20 (32.3%)
[60–69]	60 (19.8%)	60 (19.8%)	25 (16.6%)	22 (24.4%)	13 (21%)
> = 70	24 (7.9%)	24 (7.9%)	10 (6.6%)	10 (11.1%)	4 (6.4%)
BMI (kg/m2)					
<25	146 (46.6%)	146 (46.6%)	81 (51.6%)	37 (40.2%)	28 (43.8%)
[25–30[	117 (37.4%)	117 (37.4%)	57 (36.3%)	36 (39.1%)	24 (37.5%)
> = 30	50 (16.0%)	50 (16.0%)	19 (12.1%)	19 (20.6%)	12 (18.8%)
Missing	2	2	0	2	0
pCR status at surgery					
Yes	127 (40.3%)	127 (40.3%)	67 (42.7%)	39 (41.5%)	21 (32.8%)
No	188 (59.7%)	188 (59.7%)	90 (57.3%)	55 (58.5%)	43 (67.2%)
Missing	0	0	0	0	0
Presence of vascular emboli					
Yes	15 (6.5%)	15 (6.5%)	8 (7.1%)	4 (5.6%)	3 (6.5%)
No	215 (93.5%)	215 (93.5%)	104 (92.9%)	68 (94.4%)	43 (93.5%)
Missing	85	85	45	22	18
Hormonal receptors status					
ER and/or PR +	183 (60.0%)	183 (60.0%)	95 (62.1%)	51 (56.7%)	37 (59.7%)
ER and PR -	122 (40.0%)	122 (40.0%)	58 (37.9%)	39 (43.3%)	25 (40.3%)
Missing	10	10	4	4	2
SBR Grade					
SBR I	5 (1.6%)	5 (1.6%)	4 (2.6%)	0	1 (1.6%)
SBR II	139 (45.7%)	139 (45.7%)	70 (46.4%)	41 (45.6%)	28 (44.4%)
SBR III	154 (50.7%)	154 (50.7%)	74 (49%)	46 (51.1%)	34 (54.0%)
Ungradable	6 (2.0%)	6 (2.0%)	3 (2%)	3 (3.3%)	0
Missing	11	11	6	4	1
T classification					
T1c	8 (2.5%)	8 (2.5%)	3 (1.9%)	3 (3.2%)	2 (3.1%)
T2	192 (61.1%)	192 (61.1%)	102 (65.0%)	51 (54.8%)	39 (60.9%)
T3	76 (24.2%)	76 (24.2%)	32 (20.4%)	29 (31.2%)	15 (23.4%)
T4a	12 (3.8%)	12 (3.8%)	7 (4.5%)	4 (4.3%)	1 (1.6%)
T4b	4 (1.3%)	4 (1.3%)	2 (1.3%)	1 (1.1%)	1 (1.6%)
T4c	3 (1.0%)	3 (1.0%)	0	1 (1.1%)	2 (3.1%)
T4d	18 (5.7%)	18 (5.7%)	10 (6.4%)	4 (4.3%)	4 (6.2%)
TX	1 (0.3%)	1 (0.3%)	1 (0.6%)	0	0
Missing	1	1	0	1	0
N classification					
N0	112 (36.2%)	112 (36.2%)	50 (32.1%)	40 (43.9%)	22 (35.5%)
N1	147 (47.6%)	147 (47.6%)	84 (53.8%)	36 (39.6%)	27 (43.5%)
N2	22 (7.1%)	22 (7.1%)	8 (5.1%)	9 (9.9%)	5 (8.1%)
N3	3 (1.0%)	3 (1.0%)	1 (0.6%)	0	2 (3.2%)
NX	25 (8.1%)	25 (8.1%)	13 (8.3%)	6 (6.6%)	6 (9.7%)
Missing	6	6	1	3	2

*Federated = aggregation of databases 1, 2 and 3.

SD: Standard Deviation; Q1: First quartile; Q3: Third quartile; NR: Not Reported; BMI: Body Mass Index; pCR: Pathological Complete Response; ER: Estrogen Receptor; PR: Progesterone Receptor; SBR: Scarff-Bloom-Richardson; T: Tumor; N: Nodes.

For categorical variables, the results (number of observations and percentages) were exactly the same between centralized and FA. However, the privacy filter threshold (n<3 observations within a category in at least one database) was reached for 28 out of 48 categorical variables (58%). In this situation, the DataSHIELD disclosure filter restricted the analysis and aggregated results were not returned to the analyst. As a workaround, the aggregated results were obtained by tripling the source datasets to reach the threshold cell count of three and then dividing the results by three to obtain the exact count.

For continuous variables, the mean and SD were identical for the centralized and FA results. However, we observed five out of 25 continuous variables with an absolute difference of more than 5% for the median, as these statistics depend on the number of observations within each source database (even count of data points: median is the average of the two middle values, odd count of data points: median is the value of the (n+1)/2 observation).

As visualized in Table 2, more than half of the descriptive categorical variables (58%) would not have been generated in the federated report without the triplication workaround.

**Table 2:** Percentage of descriptive variables that have reached the DataSHIELD disclosure restriction filter by type of variable.

Analysis category	Type	Number of variables in the report	Number of variables that havehaving reached the disclosure restrictionlimitation filter (n<3) in DataSHIELD (%)
Descriptive statistics (all patients)	Continuous	9	0
	Categorical	29	14 (48.2%)
Descriptive statistics (by subgroup)	Continuous	16	1 (6.2%)
	Categorical	19	14 (73.7%)
Descriptive statistics (total)	Continuous	25	1 (4.0%)
	Categorical	48	28 (58.3%)

Note that the minimum and maximum values are not part of the aggregated result due to data disclosure restrictions in DataSHIELD. Only one continuous variable out of 48 was not summarized in the FA because the variable was completely missing in one of the source databases. There was no workaround for this finding due to privacy restrictions in the Datashield settings at the time of analysis.

The built-in DataSHIELD base functions were sufficient to reproduce similar descriptive statistics in the FA environment, no additional custom function was required.

### Survival analysis

Survival K-M estimates (survival probabilities at fixed year) were obtained within each database using the ds.survit function from the dsSurvival package [13]. The weighted mean by the number of patients included in each database was used as the aggregation method performed locally in R programming. Four-year survival probabilities were similar with a maximum difference of 0.3% (see Table 3).

**Table 3:** Comparison of survival probabilities for centralized and federated approaches.

Strata	Number of patient	Number of event	Survival probabilities at 4 years [95%CI]	
			Centralized	Federated
All	297	37	90.6 [85.9; 93.7]	90.6 [84.7; 97.0]
pCR results				
No pCR	175	28	88.3 [81.6; 92.7]	88.4 [79.8; 98.0]
pCR	122	9	93.8 [86.3; 97.3]	94.1 [88.2; 100.0]

CI: Confidence Interval; pCR: Pathological Complete Response.

The variability of the results between databases due to heterogeneity in the number of patients/events may affect the robustness of the aggregated results. The availability of the data and the survival trend within each database must be carefully assessed in advance.

Depending on the privacy settings of the FA, the K-M curve can be considered as a potentially disclosive output, as it can provide the exact time of occurrence of an event for a patient. At the time of the analysis, there was no built-in function in DataSHIELD to produce an aggregated non-disclosive Kaplan-Meier curve. A custom function was written to generate the exact K-M curve from the survival estimates of each source database. Since 2023, the dsSurvival2.0 package has introduced an enhanced version of the dsSurvival package that provides new privacy enhancing survival curves using locally estimated scatterplot smoothing (LOESS) method [14]. However, this package can only provide one curve per source database, not a global curve.

Univariate survival modeling was performed using the built-in ds.survival package [13]. Cox models were performed using the ds.coxph.SLMA function which includes the aggregation of the HR estimates (and standard errors) using the metafor function [15]. The effect p-values associated with HR were not calculated with this package, therefore we adapted this function to obtain the associated global effect p-value per variable for each database. The global effect p-values obtained were then aggregated using the sum of logs Fisher’s method (sumlog function from the metap package) [16].

The univariate results are shown in Table 4. The largest difference in HR was observed for N classification (N2&N3 category: centralized HR = 0.59 [0.13–2.60] vs federated HR = 1.37 [0.28–6.69]). The significance at 15% was not maintained for five up to nine variables. This difference was attributed to the heterogeneity of the source databases (the variability in database 1 was higher than in database 3, which affected the aggregated results), and to the aggregation method used to calculate the p-value (sum of logs Fisher’s method).

**Table 4:** Comparison of univariate survival results for centralized and federated approaches.

Parameter—Reference category	Category	Centralized		Federated	
		HR [95%CI]	p	HR [95%CI]	p
Age at initiation of Herceptin (years)		0.99 [0.96; 1.01]	0.325	0.99 [0.96; 1.03]	0.266
Age group (years)—<40	[40–49[	0.27 [0.09; 0.83]	0.047*	0.28 [0.09; 0.86]	0.541
	[50–59[	1.14 [0.52; 2.51]		1.27 [0.55; 2.93]	
	[60–69[	0.40 [0.14; 1.14]		0.68 [0.22; 2.07]	
	> = 70	0.51 [0.11; 2.32]		0.86 [0.18; 4.17]	
BMI (kg/m2)—<25	[25–30[	0.90 [0.43; 1.86]	0.940	0.79 [0.27; 2.32]	0.728
	> = 30	1.04 [0.41; 2.66]		1.50 [0.57; 3.97]	
T classification—T0-3	T>3	2.24 [1.04; 4.79]	0.038*	2.10 [0.96; 4.56]	0.224
N classification—N0	N1	1.30 [0.65; 2.59]	0.496	1.27 [0.62; 2.58]	0.850
	N2&N3	0.59 [0.13; 2.60]		1.37 [0.28; 6.69]	
SBR Grade—SBR I & II	SBR III	1.53 [0.79; 2.95]	0.207	1.51 [0.78; 2.93]	0.619
Presence of vascular emboli—Yes	No	0.24 [0.09; 0.64]	0.005*	0.23 [0.06; 0.90]	0.012*
HR status—ER and/or PR +	ER and PR-	1.93 [0.99; 3.77]	0.054*	1.87 [0.95; 3.69]	0.270
pCR results—pCR	No pCR	2.39 [1.13; 5.09]	0.023*	2.06 [0.75; 5.67]	0.341

BMI: Body Mass Index; pCR: Pathological Complete Response; ER: Estrogen Receptor; PR: Progesterone Receptor; SBR: Scarff-Bloom-Richardson; T: Tumor; N: Nodes.

HR [95%CI]: Hazard ratio [95% Confidence interval] obtained from a Random-Effects Model (REML) on aggregated estimates from each database.

p: Combine p-values by the sum of logs (Fisher’s) method from each database.

*p-value significant at 0.15 level.

Using a 15% threshold for the variable selection, we obtained the same final multivariate model as in the centralized analysis (Table 5). However, due to the heterogeneity of the data availability within each database (222 patients (25 events) in total with 107 (12), 70 (8) and 45 (5) per database respectively), the aggregated HR estimates were not robust enough.

**Table 5:** Comparison of multivariate survival results for centralized and federated approaches.

Parameter Reference category	Category	Centralized		Federated	
		HR [95%CI]	p	HR [95%CI]	p
Age group (years)	[40–49 [(52 / 1)	0.06 [0.01; 0.50]	0.009*	0.00 [0.00; inf.]	0.037*
<40 (41 / 9)**	[50–59 [(61 / 9)	0.90 [0.34; 2.41]		0.00 [0.00; 823.8]	
	[60–69 [(47 / 4)	0.19 [0.05; 0.67]		0.34 [0.09; 1.34]	
	> = 70 (21 / 2)	0.40 [0.08; 2.09]		0.62 [0.11; 3.47]	
T classification T0-3 (202 / 20)	T>3 (20 / 5)	4.72 [1.62; 13.8]	0.005*	inf. [0.00; inf.]	0.045*
Presence of vascular emboli Yes (15 / 5)	No (207 / 20)	0.15 [0.05; 0.45]	<0.001*	0.12 [0.04; 0.37]	0.047*
pCR results pCR (98 / 6)	No pCR (124 / 19)	3.25 [1.24; 8.55]	0.017*	3.66 [1.15; 11.6]	0.062*

pCR: Pathological Complete Response; T: Tumor.

HR [95%CI]: Hazard ratio [95% Confidence interval] obtained from a Random-Effects Model (REML) on aggregated estimates from each database.

inf.: Infinity.

p: Combine p-values by the sum of logs (Fisher’s) method from each database.

*p-value significant at 0.15 level.

**(Number of patients / Number of events).

### Logistic analysis

Univariate logistic modeling was performed using the built-in dsBaseClient package [17] in the FA environment. Logistic models were run using the ds.glmSLMA function which includes the aggregation of the OR estimates (and standard error) with the metafor function [15]. For HR estimates, a custom function was used to obtain the associated global effect p-value for each database by variable. The global effect p-values were then also aggregated using the sum of logs Fisher’s method (sumlog function from the metap package) [16]. The univariate results are shown in Table 6.

**Table 6:** Comparison of univariate logistic results for centralized and federated approaches.

Parameter—Reference category	Category	Centralized		Federated	
		OR [95%CI]	p	OR [95%CI]	p
Age at initiation of Herceptin (years)		1.00 [0.98; 1.02]	0.997	1.00 [0.98; 1.02]	0.735
Age group (years)—<40	[40–49[	0.87 [0.43; 1.78]	0.701	0.86 [0.41; 1.77]	0.900
	[50–59[	0.85 [0.43; 1.70]		0.86 [0.43; 1.72]	
	[60–69[	0.70 [0.33; 1.50]		0.74 [0.34; 1.60]	
	> = 70	1.54 [0.59; 4.13]		1.54 [0.57; 4.13]	
BMI (kg/m2)—<25	[25–30[	0.98 [0.60; 1.61]	0.946	0.98 [0.60; 1.61]	0.118*
	> = 30	0.51 [0.25; 1.01]		0.56 [0.15; 2.03]	
T classification—T0-3	T>3	0.79 [0.38; 1.60]	0.526	0.82 [0.37; 1.83]	0.404
N classification—N0	N1	1.09 [0.66; 1.81]	0.749	0.86 [0.31; 2.36]	0.119*
	N2&N3	2.12 [0.89; 5.20]		2.06 [0.85; 4.96]	
SBR Grade—SBR I & II	SBR III	1.03 [0.65; 1.63]	0.912	0.94 [0.48; 1.88]	0.220
Presence of vascular emboli—Yes	No	0.87 [0.30; 2.57]	0.797	0.88 [0.30; 2.53]	0.993
HR status—ER and/or PR +	ER and PR-	1.11 [0.69; 1.76]	0.668	1.08 [0.62; 1.86]	0.436

BMI: Body Mass Index; ER: Estrogen Receptor; PR: Progesterone Receptor; SBR: Scarff-Bloom-Richardson; T: Tumor; N: Nodes.

OR [95%CI]: Odds Ratio [95% Confidence Interval] obtained from a Random-Effects Model (REML) on aggregated estimates from each database.

p: Combine p-values by the sum of logs (Fisher’s) method from each database.

*p-value significant at 0.15 level.

The largest difference observed for OR was for N classification (N1 category: centralized OR = 1.09 [0.66–1.81] vs federated HR = 0.86 [0.31–2.36]). Significance at 15% was not maintained in the FA for 2 to 8 variables compared to the centralized analysis, this difference was attributed to the heterogeneity of the source databases (more variability in database 1 than in database 3) and the aggregation method of the p-value (sum of logs Fisher’s method). No additional variable was selected for the multivariate model at the 15% threshold.

### Correlation matrix

**R**elationships between two categorical variables were assessed using either the Chi-square test or Fisher’s exact test. These tests are based on the aggregated counts, therefore no DataSHIELD function was required, and the results obtained in FA were exactly equivalent to the centralized results.

Relationships between continuous and categorical variables were assessed using the global effect p-value from ANOVA. This p-value was not available in the built-in DataSHIELD function (ds.glmSLMA). To address this, we customized the function to retrieve the global effect p-value for each database. These global effect p-values were then aggregated using the sum of logs Fisher’s method [16]. The same level of relationship as in the centralized environment was observed at the 5% threshold (Table 7).

**Table 7:** Correlation matrix for centralized and federated approaches.

		Federated								
	*Variables*	Age (years) *n*	Age group *c*	BMI group *c*	T class *c*	N class *c*	SBR grade *c*	V.E. *c*	H.R. *c*	pCR *c*
**Centralized**	Age (years) *n*			**<0.001**	0.2679	**<0.001**	0.8403	0.3867	**0.0059**	0.7409
	Age group *c*			**<0.001**	**0.0260**	**0.0220**	0.9471	0.9045	**0.0413**	0.5857
	BMI group *c*	**<0.001**	**<0.001**		**0.0142**	0.4921	**0.0356**	0.8036	0.0920	0.1418
	T class. *c*	0.0896	**0.0205**	**0.0142**		**0.0456**	1	0.3723	0.1132	0.6482
	N class. *c*	**<0.001**	**0.0185**	0.4888	**0.0455**		0.0819	0.1664	**0.0021**	0.2264
	SBR grade *c*	0.8627	0.9471	**0.0356**	1	0.0819		0.285	**0.0110**	1
	V.E. *c*	0.7479	0.9075	0.8201	0.3723	0.1499	0.3014		0.6001	1
	H.R. *c*	**0.0053**	**0.0413**	0.0920	0.1132	**0.0021**	**0.0110**	0.7476		0.7568
	pCR *c*	0.9967	0.5857	0.1418	0.6482	0.2264	1	1	0.7568	

BMI: Body Mass Index; H.R.: Hormonal Receptor; SBR: Scarff-Bloom-Richardson; T class.: Tumor classification; N class.: Nodes classification.; V.E.: Vascular Emboli; pCR: Pathological Complete Response.

bold cell: P-value significant at 0.05 level.

*n*: Numerical (continuous) variable type

*c*: Categorical variable type.

- Between quantitative and qualitative variables: Anova global effect p-value displayed in the table.

- Between qualitative variables: Chi² test has been used when all expected counts are > = 5. Otherwise, the Fisher exact test has been used. p-value displayed in the table.

Note: P-value for Fisher exact test not exactly similar between both approaches because different seed number used.

## Limitation

This proof-of-concept of a federated approach was implemented using only a single synthetic real-world data representing a non-interventional study, divided into three virtual healthcare organizations of different sizes in terms of number of patients. This setting was an ideal federated environment: limited number of sources and data already harmonized. This proof-of-concept should be generalized to a broader federated real-world scenario, representative of practical applications, where data privacy, availability and harmonization should be anticipated and mitigated. The availability of the centralized results was also helpful in carrying out the FA programming, which will not be the case in a real-world setting. It would be interesting to evaluate how FA performs under different scenarios in terms of data volumes, data types, level of data quality levels and different privacy preserving settings.

Federated learning using more complex models (deep learning, neural networks, …) was also outside the scope of our research, as this was not originally planned. These approaches could be investigated using the same methodology of this research.

## Discussion

Our research showed a practical implementation of an FA environment in a proof-of-concept configuration and its use. The following key insights can be shared for future implementation of such an approach:

- The implementation of a federated environment in real-world setting needs to be anticipated and discussions with the participating healthcare organizations need to be initiated in advance in order to fit the approach within the existing infrastructure and privacy requirements.
- The level of data disclosure prevention to be set in the FA should be agreed with all participants in accordance with technical and local regulatory requirements, to establish the right balance between privacy and accuracy (e.g., for small cohorts, a trade-off between privacy and feasibility needs to be made).
- Establish a governance framework to control the access to the FA environment, setting appropriate access for each data owner and analyst while maintaining the privacy of the individual data.
- Establish a governance framework for the creation of custom DataSHIELD functions (data server and client machines) to ensure the security of the function and the non-disclosure of sensitive information. All custom functions must be tested and validated by all data custodians prior to local deployment in the FA environment.
- Participating healthcare organizations may use different data structures. To avoid statistical inaccuracies, data standardization should be performed in advance to harmonize the selected data between the FA network organizations (without data transfer). Data quality measurement can also be performed in a federated architecture using not disclosive data quality measurement functions.
- The variability of the data between individual source databases may affect the accuracy of the FA results. A data review to assess the quality and disposition of each database should be performed in order to minimize the potential statistical bias and understand the data. For organizations with a small number of patients, this means either relaxing FA restriction rules or pooling data before performing the FA analysis (i.e. a hybrid centralized-federated solution).
- It is recommended to prepare a federated statistical analysis plan before performing the analyses. The document should describe the source data (number of databases, number of patients per database and type of variable), the analysis to be performed and the aggregation methods to be used to ensure the feasibility and accuracy of the analysis. An evaluation of the standard/custom functions to be used according to the data structure and DataSHIELD capabilities at the time of the analysis must also be performed.
- As for a centralized approach, variable derivations to support further analysis requirements should be performed prior to using any of the analytical functions of DataSHIELD.

## Conclusions

The DataSHIELD open-source solution was used to generate a federated statistical report from three local source databases with different sample sizes but with identical structure (variable and format). In our proof-of-concept situation, the data was partitioned horizontally, with the same data structure across all source databases but on different patients. Common data transformations and statistical analyses were reproduced using DataSHIELD programming and compared with the centralized report generated from the raw database. Both analyses were performed using R-based programming.

All analyses were successfully reproduced using built-in or custom DataSHIELD functions. The FA results were identical to the raw results in terms of descriptive statistics, except for some differences in positional measures (quartiles). The FA estimates of the univariate models were aligned with those from the centralized results, but a loss of accuracy was observed for the multivariate model due to source database variability. The accuracy of the FA results is related to the statistical aggregation method used and the number of data points within each source database. Centers with fewer patients will result in more federated privacy restrictions and a greater loss of accuracy than in a centralized setting.

The capability and accuracy of common data manipulation and statistical analysis was satisfactory with DataSHIELD. The flexibility of the tool (ability to develop new functions) allows a variety of analyses to be carried out while maintaining the privacy of individual sensitive data, no blocking points were identified. The DataSHIELD forum was a practical source of information and support. In order to find the right balance between privacy and accuracy of the analysis, the privacy requirements should be established before starting the analysis. The FA approach is a good alternative when a centralized approach is not feasible due to data access and/or data sharing issues.

This approach is suitable for real-world research using multiple data sources (either at site level or at national cohort level), as long as the data are harmonized beforehand. For prospective studies, it may be preferable to use an electronic Case Report Form (eCRF) to ensure harmonization of data collection, while still using the FA approach for statistical analysis. Finally, the FA approach still requires the necessary research approvals, depending on the regulations in the countries involved in the research.

## Supporting information

S1 Table R functions used for federated and centralized programming. (TIF)

S2 Table Aggregation methods used for federated analysis. (TIF)

S1 File Statistical report centralized. (DOCX)

S2 File Statistical report federated. (DOCX)

S3 File Rmd code data import federated. (TXT)

S4 File Rmd code data management federated. (TXT)

S5 File Rmd code descriptive stat federated. (TXT)

S6 File Rmd code correlations federated. (TXT)

S7 File Rmd code survival federated. (TXT)

S8 File Rmd code logistic federated. (TXT)

S9 File R code dataset derivation centralized. (TXT)

S10 File R code generate analysis centralized. (TXT)

## References

[1] Rosenbaum L. Bridging the Data-Sharing Divide—Seeing the Devil in the Details, Not the Other Camp. N Engl J Med. 2017 Jun 8;376(23):2201–2203. doi: 10.1056/NEJMp1704482 Epub 2017 Apr 26. . 28445080

[2] E. Parliament, C. of European Union, Regulation (eu) 2016/679 of the european parliament and council (2016). https://eur-lex.europa.eu/eli/reg/2016/679/oj.

[3] Edemekong, Peter F.; Annamaraju, Pavan; Haydel, Micelle J. (2023). Health Insurance Portability and Accountability Act, StatPearls, Treasure Island (FL): StatPearls Publishing. 29763195

[4] Templ M., Sariyar M. A systematic overview on methods to protect sensitive data provided for various analyses. Int. J. Inf. Secur. 21, 1233–1246 (2022). Available from: doi: 10.1007/s10207-022-00607-5

[5] Gaye A, Marcon Y, Isaeva J, LaFlamme P, Turner A, Jones EM, et al. DataSHIELD: taking the analysis to the data, not the data to the analysis. Int J Epidemiol. 2014 Dec;43(6):1929–44. doi: 10.1093/ije/dyu188 Epub 2014 Sep 26. 25261970 PMC4276062

[6] DataSHIELD list of functions. https://data2knowledge.atlassian.net/wiki/spaces/DSDEV/overview.

[7] DataSHIELD community packages. https://www.datashield.org/help/community-packages.

[8] DataSHIELD disclosure controls. https://data2knowledge.atlassian.net/wiki/spaces/DSDEV/pages/714768398/Disclosure+control.

[9] Cox DR (1972). Regression models and life-tables (with discussion). Journal of the Royal Statistical Society. Series B (Methodological) 34 (2), 187–220.

[10] Cox DR. The regression analysis of binary sequences. Journal of the Royal Statistical Society, Series B. 1958;20:215–242.

[11] Dragan, I., Sparsø, T., Kuznetsov, D., Slieker, R. & Ibberson, M. dsSwissKnife: An R package for federated data analysis. 10.1101/2020.11.17.386813 (2020).

[12] ds-Helper package. https://github.com/lifecycle-project/ds-helper.

[13] Banerjee S, Sofack GN, Papakonstantinou T, Avraam D, Burton P, Zöller D, et al. dsSurvival: Privacy preserving survival models for federated individual patient meta-analysis in DataSHIELD. BMC Res Notes. 2022 Jun 3;15(1):197. doi: 10.1186/s13104-022-06085-1 35659747 PMC9166323

[14] Banerjee S, Bishop TRP. dsSurvival 2.0: privacy enhancing survival curves for survival models in the federated DataSHIELD analysis system. BMC Res Notes. 2023 Jun 6;16(1):98. doi: 10.1186/s13104-023-06372-5 37280717 PMC10243006

[15] Schwarzer G. R. (2007). An R package for meta‐analysis. R News, 7, 40–45. Available from: http://www.metafor-project.org/doku.php.

[16] Dewey M (2023). metap: Meta-Analysis of Significance Values. R package version 1.9. https://CRAN.R-project.org/package=metap.

[17] Developers D (2023). dsBaseClient: DataSHIELD Client Functions. R package version 6.3.0.

