# Supplementary figures and images for: Capability and accuracy of usual statistical analyses in a real-world setting using a federated approach
Source: PLoS One. 2024 Nov 14;19(11):e0312697. doi: 10.1371/journal.pone.0312697 (PMC11563485; doi:10.1371/journal.pone.0312697)

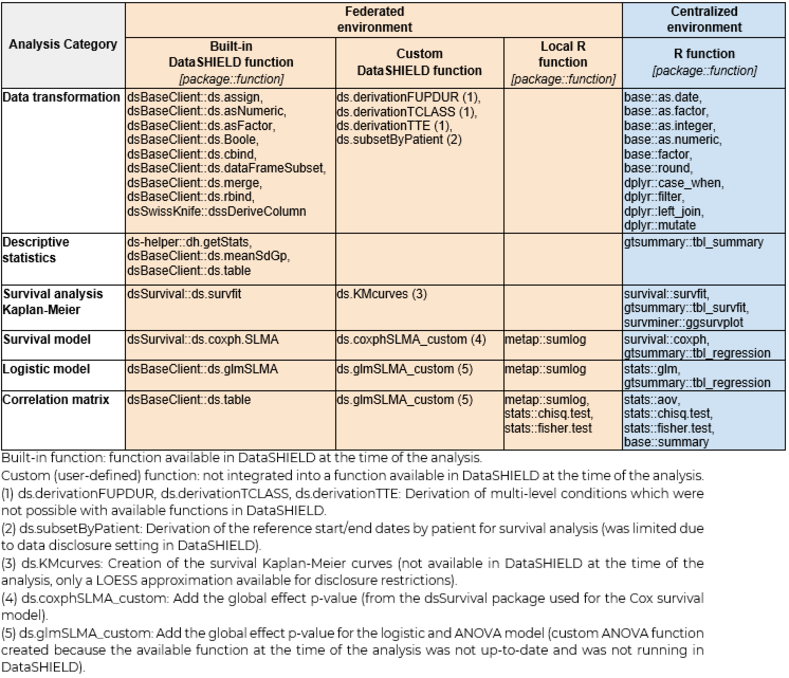

Supplement: S1 Table — (TIF) [file pone.0312697.s001.tif]

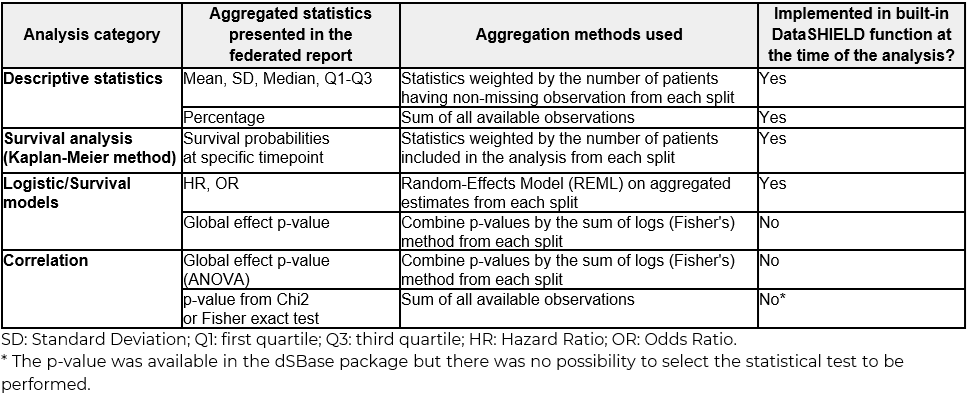

Supplement: S2 Table — (TIF) [file pone.0312697.s002.tif]
