# Supplementary material for: Capability and accuracy of usual statistical analyses in a real-world setting using a federated approach
Source: PLoS One. 2024 Nov 14;19(11):e0312697. doi: 10.1371/journal.pone.0312697 (PMC11563485; doi:10.1371/journal.pone.0312697)

KADOR PoC Anonymisation – Analysis report on the avatarization anonymized data

A FRENCH RETROSPECTIVE STUDY DESCRIBING THE EPIDEMIOLOGY AND THE THERAPEUTIC MANAGEMENT OF PATIENTS TREATED BY HERCEPTIN® BASED NEOADJUVANT TREATMENT FOR HER2-POSITIVE EARLY BREAST CANCER

Laetitia Vinet

2022-10-25

Table of Contents

[**1 Analysis of study conduct 1**](#_heading=)

[1.1 Patient Disposition 1](#_heading=)

[Table 1.1.1 Summary of patient disposition - Full Analysis Set Population 1](#_heading=)

[Table 1.1.2 Latest news - Among patients not being followed in the site - Full Analysis Set Population 2](#_heading=)

[Table 1.1.3 Cause of death - Among dead patients - Full Analysis Set Population 4](#_heading=)

[Table 1.1.4 Time from diagnostic to progression - Among patients having experienced progression of the disease since the beginning of adjuvant therapy - Full Analysis Set Population 5](#_heading=)

[Table 1.1.5 Dates available - Full Analysis Set Population 6](#_heading=)

[**2 Baseline characteristics 8**](#_heading=)

[2.1 Demographics and baseline disease characteristics 8](#_heading=)

[Table 2.1.1 Summary of demographics and baseline disease characteristics - Full Analysis Set Population 8](#_heading=)

[Table 2.1.2 Summary of demographics and baseline disease characteristics by pCR result - Full Analysis Set Population 13](#_heading=)

[**3 Surgery and pCR 18**](#_heading=)

[3.1 Surgery 18](#_heading=)

[3.1.1 Summary of surgery - Among patients with at least one surgery - Full Analysis Set Population 18](#_heading=)

[3.2 pCR 19](#_heading=)

[3.2.1 Summary of pCR - Full Analysis Set Population 19](#_heading=)

[**4 Adjuvant treatments 21**](#_heading=)

[Table 4.1 Summary of adjuvant treatments - Among subjects with at least one adjuvant treatments - Full Analysis Set Population 21](#_heading=)

[Table 4.2 Summary of adjuvant treatments by adjuvant treatment - Among subjects with at least one adjuvant treatments - Full Analysis Set Population 22](#_heading=)

[Table 4.3 Time between surgery and adjuvant treatment - Among subjects with at least one adjuvant treatments - Full Analysis Set Population 37](#_heading=)

[Table 4.4 Summary of adjuvant treatments by pCR status - Among subjects with at least one adjuvant treatments - Full Analysis Set Population 38](#_heading=)

[Table 4.5 Summary of adjuvant treatments by adjuvant treatment by pCR status - Among subjects with at least one adjuvant treatments - Full Analysis Set Population 39](#_heading=)

[Table 4.6 Time between surgery and adjuvant treatment by pCR status - Among subjects with at least one adjuvant treatments - Full Analysis Set Population 1](#_heading=)

[**5 Efficacy Analyses 1**](#_heading=)

[5.1 Time to event analyses 1](#_heading=)

[Table 5.1.1 Summary of time from herceptin adjuvant treatment to PFS, overall and by pCR result - Kaplan-Meier estimation - Among subjects with herceptin adjuvant treatment start date available - Full Analysis Set Population 1](#_heading=)

[Table 5.1.2 Survival probabilities of time from herceptin adjuvant treatment to PFS, overall and by pCR result - Kaplan-Meier estimation - Among subjects with herceptin adjuvant treatment start date available - Full Analysis Set Population 1](#_heading=)

[Table 5.1.3 Summary of time from herceptin adjuvant treatment to PFS - Kaplan-Meier curve - Among subjects with herceptin adjuvant treatment start date available - Full Analysis Set Population 1](#_heading=)

[Table 5.1.4 Summary of time from herceptin adjuvant treatment to PFS by pCR result - Kaplan-Meier curve - Among subjects with herceptin adjuvant treatment start date available - Full Analysis Set Population 1](#_heading=)

[**6 Exploratory Analyses 1**](#_heading=)

[6.1 Predictive factors for PFS 1](#_heading=)

[Table 6.1.1 PFS - Univariate Cox proportional hazard analysis - Among subjects with herceptin adjuvant treatment start date available - Full Analysis Set Population 1](#_heading=)

[Table 6.1.2 PFS - Multivariate Cox proportional hazard analysis - Among subjects with herceptin adjuvant treatment start date available - Full Analysis Set Population 1](#_heading=)

[6.2 Predictive factors for pCR result 1](#_heading=)

[Table 6.2.1 pCR result - Univariate analysis - Full Analysis Set Population 1](#_heading=)

[Table 6.2.2 pCR result - Multivariate analysis - Full Analysis Set Population 1](#_heading=)

[6.3 Predictive factors for PFS and pCR result 1](#_heading=)

[Table 6.3.1 Correlation matrix - Full Analysis Set Population 1](#_heading=)

[Figure 6.3.2 Correlation coefficient matrix - Full Analysis Set Population 1](#_heading=)

# 1 Analysis of study conduct

## 1.1 Patient Disposition

### Table 1.1.1 Summary of patient disposition - Full Analysis Set Population

| Characteristic | All (N = 315) |
| --- | --- |
| Follow-up duration (years) |  |
| Nobs | 226 |
| Mean (SD) | 4.55 (0.58) |
| Median (Q1;Q3) | 4.7 (4.4; 4.9) |
| Min - Max | 1.1, 5.3 |
| Missing | 89 |
| Is the patient still being followed in the site (as of December 31, 2018)?, n/N (%) |  |
| Yes | 251/306 (82.0%) |
| No | 55/306 (18.0%) |
| Missing | 9 |
| Time from diagnostic to surgery (months) |  |
| Nobs | 257 |
| Mean (SD) | 6.98 (2.20) |
| Median (Q1;Q3) | 6.6 (6.0; 7.2) |
| Min - Max | 4.5, 26.9 |
| Missing | 58 |
| Has there been any progression of the disease since the beginning of adjuvant therapy, n/N (%) |  |
| Yes | 39/304 (12.8%) |
| No | 265/304 (87.2%) |
| Missing | 11 |
| Follow-up duration (years) = (Last consultation date/Death date – initial diagnosis date of breast cancer + 1) / 365.25 | |
| Time from diagnostic to surgery (months) = (Surgery date – initial diagnosis date of breast cancer) / (365.25/12) | |

### Table 1.1.2 Latest news - Among patients not being followed in the site - Full Analysis Set Population

| Characteristic | All (N = 55) |
| --- | --- |
| Latest news |  |
| The patient is dead | 15/52 (28.8%) |
| The patient is lost to follow-up | 35/52 (67.3%) |
| Other reason | 2/52 (3.8%) |
| Missing | 3 |

### Table 1.1.3 Cause of death - Among dead patients - Full Analysis Set Population

| Characteristic | All (N = 15) |
| --- | --- |
| Cause of death |  |
| Disease progression | 11/13 (84.6%) |
| Other reason | 2/13 (15.4%) |
| Missing | 2 |

### Table 1.1.4 Time from diagnostic to progression - Among patients having experienced progression of the disease since the beginning of adjuvant therapy - Full Analysis Set Population

| Characteristic | All (N = 39) |
| --- | --- |
| Time from diagnostic to progression (years) |  |
| Nobs | 26 |
| Mean (SD) | 2.43 (1.01) |
| Median (Q1;Q3) | 2.3 (1.7; 3.1) |
| Min - Max | 0.9, 4.4 |
| Missing | 13 |
| Time from diagnostic to progression (years) = (Date of the first progression of the disease – initial diagnosis date of breast cancer) / 365.25 | |

### Table 1.1.5 Dates available - Full Analysis Set Population

| Characteristic | All (N = 315) |
| --- | --- |
| Birth date available |  |
| Yes | 315/315 (100.0%) |
| No | 0/315 (0.0%) |
| Initial diagnosis date of breast cancer available |  |
| Yes | 259/315 (82.2%) |
| No | 56/315 (17.8%) |
| Surgery date available |  |
| Yes | 304/315 (96.5%) |
| No | 11/315 (3.5%) |
| Date of last consultation available among patients still being followed in the site (as of December 31, 2018) |  |
| Yes | 251/251 (100.0%) |
| No | 0/251 (0.0%) |
| Date of the first progression of the disease available among patients having experienced progression of the disease since the beginning of adjuvant therapy |  |
| Yes | 34/39 (87.2%) |
| No | 5/39 (12.8%) |
| Death date available among dead patients |  |
| Yes | 14/15 (93.3%) |
| No | 1/15 (6.7%) |

# 2 Baseline characteristics

## 2.1 Demographics and baseline disease characteristics

### Table 2.1.1 Summary of demographics and baseline disease characteristics - Full Analysis Set Population

| Characteristic | All (N = 315) |
| --- | --- |
| Age at adjuvant treatment initiation of Herceptin (years) |  |
| Nobs | 303 |
| Mean (SD) | 52.23 (11.82) |
| Median (Q1;Q3) | 52.0 (43.0; 60.5) |
| Min - Max | 29, 77 |
| Missing | 12 |
| Age group (years), n/N (%) |  |
| <40 | 53/303 (17.5%) |
| [40 - 49] | 75/303 (24.8%) |
| [50 - 59] | 91/303 (30.0%) |
| [60 - 69] | 60/303 (19.8%) |
| >=70 | 24/303 (7.9%) |
| Missing | 12 |
| BMI (kg/m2), n/N (%) |  |
| <25 | 146/313 (46.6%) |
| [25 - 30[ | 117/313 (37.4%) |
| >=30 | 50/313 (16.0%) |
| Missing | 2 |
| Professional situation, n/N (%) |  |
| Worker | 162/274 (59.1%) |
| Jobless person | 68/274 (24.8%) |
| Data not found | 44/274 (16.1%) |
| Missing | 41 |
| Weight (kg) |  |
| Nobs | 314 |
| Mean (SD) | 67.88 (11.49) |
| Median (Q1;Q3) | 66.0 (60.0; 74.0) |
| Min - Max | 49, 121 |
| Missing | 1 |
| Height(cm) |  |
| Nobs | 313 |
| Mean (SD) | 162.99 (4.27) |
| Median (Q1;Q3) | 163.0 (160.0; 165.0) |
| Min - Max | 150, 178 |
| Missing | 2 |
| Weight at initiation of adjuvant therapy (kg) |  |
| Nobs | 265 |
| Mean (SD) | 67.34 (10.87) |
| Median (Q1;Q3) | 66.0 (60.0; 72.0) |
| Min - Max | 45, 118 |
| Missing | 50 |
| Classification T, n/N (%) |  |
| T1c | 8/314 (2.5%) |
| T2 | 192/314 (61.1%) |
| T3 | 76/314 (24.2%) |
| T4a | 12/314 (3.8%) |
| T4b | 4/314 (1.3%) |
| T4c | 3/314 (1.0%) |
| T4d | 18/314 (5.7%) |
| TX | 1/314 (0.3%) |
| Missing | 1 |
| Classification N, n/N (%) |  |
| N0 | 112/309 (36.2%) |
| N1 | 147/309 (47.6%) |
| N2 | 22/309 (7.1%) |
| N3 | 3/309 (1.0%) |
| NX | 25/309 (8.1%) |
| Missing | 6 |
| Histology at the initial diagnosis, n/N (%) |  |
| Invasive ductal carcinoma | 291/306 (95.1%) |
| Invasive lobular carcinoma | 9/306 (2.9%) |
| Other | 6/306 (2.0%) |
| Missing | 9 |
| Presence of vascular emboli, n/N (%) |  |
| Yes | 15/230 (6.5%) |
| No | 215/230 (93.5%) |
| Missing | 85 |
| SBR grade, n/N (%) |  |
| SBR I | 5/304 (1.6%) |
| SBR II | 139/304 (45.7%) |
| SBR III | 154/304 (50.7%) |
| Ungradable | 6/304 (2.0%) |
| Missing | 11 |
| Number of lymph nodes invaded |  |
| Nobs | 221 |
| Mean (SD) | 0.86 (1.11) |
| Median (Q1;Q3) | 1.0 (0.0; 1.0) |
| Min - Max | 0, 6 |
| Missing | 94 |
| Estrogen receptors, n/N (%) |  |
| positive | 167/306 (54.6%) |
| negative | 139/306 (45.4%) |
| not assessable | 0/306 (0.0%) |
| Missing | 9 |
| Progesterone receptors, n/N (%) |  |
| positive | 107/305 (35.1%) |
| negative | 198/305 (64.9%) |
| not assessable | 0/305 (0.0%) |
| Missing | 10 |
| Hormonal receptors status, n/N (%) |  |
| ER and/or PR + | 183/305 (60.0%) |
| ER and PR - | 122/305 (40.0%) |
| Missing | 10 |

### Table 2.1.2 Summary of demographics and baseline disease characteristics by pCR result - Full Analysis Set Population

| Characteristic | pCR (N = 127) | No pCR (N = 188) |
| --- | --- | --- |
| Age at adjuvant treatment initiation of Herceptin (years) |  |  |
| Nobs | 123 | 180 |
| Mean (SD) | 52.23 (12.26) | 52.23 (11.54) |
| Median (Q1;Q3) | 52.0 (44.0; 61.5) | 53.0 (43.0; 60.0) |
| Min - Max | 29, 77 | 29, 76 |
| Missing | 4 | 8 |
| Age group (years), n/N (%) |  |  |
| <40 | 23/123 (18.7%) | 30/180 (16.7%) |
| [40 - 49] | 30/123 (24.4%) | 45/180 (25.0%) |
| [50 - 59] | 36/123 (29.3%) | 55/180 (30.6%) |
| [60 - 69] | 21/123 (17.1%) | 39/180 (21.7%) |
| >=70 | 13/123 (10.6%) | 11/180 (6.1%) |
| Missing | 4 | 8 |
| BMI (kg/m2), n/N (%) |  |  |
| <25 | 63/127 (49.6%) | 83/186 (44.6%) |
| [25 - 30[ | 50/127 (39.4%) | 67/186 (36.0%) |
| >=30 | 14/127 (11.0%) | 36/186 (19.4%) |
| Missing | 0 | 2 |
| Professional situation, n/N (%) |  |  |
| Worker | 72/111 (64.9%) | 90/163 (55.2%) |
| Jobless person | 25/111 (22.5%) | 43/163 (26.4%) |
| Data not found | 14/111 (12.6%) | 30/163 (18.4%) |
| Missing | 16 | 25 |
| Weight (kg) |  |  |
| Nobs | 127 | 187 |
| Mean (SD) | 66.38 (9.98) | 68.89 (12.33) |
| Median (Q1;Q3) | 65.0 (59.0; 71.5) | 66.0 (61.0; 75.0) |
| Min - Max | 51, 108 | 49, 121 |
| Missing | 0 | 1 |
| Height(cm) |  |  |
| Nobs | 127 | 186 |
| Mean (SD) | 163.00 (3.99) | 162.99 (4.46) |
| Median (Q1;Q3) | 163.0 (160.0; 166.0) | 163.0 (160.0; 165.0) |
| Min - Max | 153, 172 | 150, 178 |
| Missing | 0 | 2 |
| Weight at initiation of adjuvant therapy (kg) |  |  |
| Nobs | 108 | 157 |
| Mean (SD) | 66.81 (9.66) | 67.70 (11.65) |
| Median (Q1;Q3) | 66.0 (60.0; 71.2) | 66.0 (61.0; 72.0) |
| Min - Max | 51, 108 | 45, 118 |
| Missing | 19 | 31 |
| Classification T, n/N (%) |  |  |
| T1c | 4/126 (3.2%) | 4/188 (2.1%) |
| T2 | 75/126 (59.5%) | 117/188 (62.2%) |
| T3 | 33/126 (26.2%) | 43/188 (22.9%) |
| T4a | 8/126 (6.3%) | 4/188 (2.1%) |
| T4b | 2/126 (1.6%) | 2/188 (1.1%) |
| T4c | 0/126 (0.0%) | 3/188 (1.6%) |
| T4d | 3/126 (2.4%) | 15/188 (8.0%) |
| TX | 1/126 (0.8%) | 0/188 (0.0%) |
| Missing | 1 | 0 |
| Classification N, n/N (%) |  |  |
| N0 | 42/122 (34.4%) | 70/187 (37.4%) |
| N1 | 58/122 (47.5%) | 89/187 (47.6%) |
| N2 | 12/122 (9.8%) | 10/187 (5.3%) |
| N3 | 2/122 (1.6%) | 1/187 (0.5%) |
| NX | 8/122 (6.6%) | 17/187 (9.1%) |
| Missing | 5 | 1 |
| Histology at the initial diagnosis, n/N (%) |  |  |
| Invasive ductal carcinoma | 116/124 (93.5%) | 175/182 (96.2%) |
| Invasive lobular carcinoma | 4/124 (3.2%) | 5/182 (2.7%) |
| Other | 4/124 (3.2%) | 2/182 (1.1%) |
| Missing | 3 | 6 |
| Presence of vascular emboli, n/N (%) |  |  |
| Yes | 7/100 (7.0%) | 8/130 (6.2%) |
| No | 93/100 (93.0%) | 122/130 (93.8%) |
| Missing | 27 | 58 |
| SBR grade, n/N (%) |  |  |
| SBR I | 2/122 (1.6%) | 3/182 (1.6%) |
| SBR II | 56/122 (45.9%) | 83/182 (45.6%) |
| SBR III | 63/122 (51.6%) | 91/182 (50.0%) |
| Ungradable | 1/122 (0.8%) | 5/182 (2.7%) |
| Missing | 5 | 6 |
| Number of lymph nodes invaded |  |  |
| Nobs | 94 | 127 |
| Mean (SD) | 0.73 (0.86) | 0.95 (1.25) |
| Median (Q1;Q3) | 1.0 (0.0; 1.0) | 1.0 (0.0; 1.0) |
| Min - Max | 0, 4 | 0, 6 |
| Missing | 33 | 61 |
| Estrogen receptors, n/N (%) |  |  |
| positive | 61/124 (49.2%) | 106/182 (58.2%) |
| negative | 63/124 (50.8%) | 76/182 (41.8%) |
| not assessable | 0/124 (0.0%) | 0/182 (0.0%) |
| Missing | 3 | 6 |
| Progesterone receptors, n/N (%) |  |  |
| positive | 45/123 (36.6%) | 62/182 (34.1%) |
| negative | 78/123 (63.4%) | 120/182 (65.9%) |
| not assessable | 0/123 (0.0%) | 0/182 (0.0%) |
| Missing | 4 | 6 |
| Hormonal receptors status, n/N (%) |  |  |
| ER and/or PR + | 72/123 (58.5%) | 111/182 (61.0%) |
| ER and PR - | 51/123 (41.5%) | 71/182 (39.0%) |
| Missing | 4 | 6 |

# 3 Surgery and pCR

## 3.1 Surgery

### 3.1.1 Summary of surgery - Among patients with at least one surgery - Full Analysis Set Population

| Characteristic | All (N = 315) |
| --- | --- |
| At least one Surgery* |  |
| Axillary curage | 249 (79%) |
| Mastectomy | 169 (53.7%) |
| Conservative surgery | 146 (46.3%) |
| Sentinel Ganglion | 53 (16.8%) |
| * One patient can have reported several surgery types | |

## 3.2 pCR

### 3.2.1 Summary of pCR - Full Analysis Set Population

| Characteristic | All (N = 315) |
| --- | --- |
| pCR results* |  |
| pCR | 127/315 (40.3%) |
| No pCR | 188/315 (59.7%) |
| Missing | 0 |
| Absence of invasive and in situ residues in the breast and in the lymph nodes |  |
| Yes | 107/256 (41.8%) |
| No | 149/256 (58.2%) |
| Missing | 59 |
| Absence of invasive residues in the breast and lymph nodes, regardless of the presence of ductal carcinoma in situ |  |
| Yes | 108/256 (42.2%) |
| No | 148/256 (57.8%) |
| Missing | 59 |
| Classification Chevallier |  |
| Grade 1 | 1/4 (25.0%) |
| Grade 2 | 1/4 (25.0%) |
| Grade 3 | 2/4 (50.0%) |
| Missing | 311 |
| Classification Sataloff T |  |
| TA | 33/51 (64.7%) |
| TB | 15/51 (29.4%) |
| TC | 3/51 (5.9%) |
| Missing | 264 |
| Classification Sataloff N |  |
| NA | 24/51 (47.1%) |
| NB | 23/51 (45.1%) |
| NC | 4/51 (7.8%) |
| Missing | 264 |
| Classification RCB |  |
| RCB-II | 1/1 (100.0%) |
| Missing | 314 |
| * pCR results = pCR if ypT0/Tis ypN0 is ticked Yes OR, Grade 1 or Grade 2 are ticked for Classification Chevallier OR, TA and NA are ticked for Classification Sataloff OR, RCB0 is ticked for Classification RCB | |

# 4 Adjuvant treatments

## Table 4.1 Summary of adjuvant treatments - Among subjects with at least one adjuvant treatments - Full Analysis Set Population

| Characteristic | All (N = 305) |
| --- | --- |
| At least one Adjuvant Treatment* |  |
| Trastuzumab (Herceptin) | 304 (99.7%) |
| Tamoxifene | 81 (26.6%) |
| Letrozole | 40 (13.1%) |
| Anastrozole | 20 (6.6%) |
| Exemestane | 5 (1.6%) |
| Other hormonotherapy 1 | 3 (1%) |
| Other | 2 (0.7%) |
| * One patient can have reported several adjuvant treatment types | |

## Table 4.2 Summary of adjuvant treatments by adjuvant treatment - Among subjects with at least one adjuvant treatments - Full Analysis Set Population

| Characteristic | All |
| --- | --- |
| Anastrozole :  - Duration (months) |  |
| Nobs | 1 |
| Mean (SD) | 15.80 (NA) |
| Median (Q1;Q3) | 15.8 (15.8; 15.8) |
| Min - Max | 15.8, 15.8 |
| Missing | 19 |
| - Administration frequency |  |
| Nobs | 16 |
| Mean (SD) | 4.00 (0.00) |
| Median (Q1;Q3) | 4.0 (4.0; 4.0) |
| Min - Max | 4.0, 4.0 |
| Missing | 4 |
| - Maintenance dose (cycle) |  |
| Nobs | 0 |
| Mean (SD) | NA (NA) |
| Median (Q1;Q3) | NA (NA; NA) |
| Min - Max | NA, NA |
| Missing | 20 |
| - Maintenance dose (cycle mg/kg or mg) |  |
| Nobs | 6 |
| Mean (SD) | 11.33 (9.50) |
| Median (Q1;Q3) | 11.5 (3.0; 20.0) |
| Min - Max | 2.0, 20.0 |
| Missing | 14 |
| - Route of administration, n/N (%) | 0/0 (NA%) |
| Missing | 20 |
| - Number of cycles completed |  |
| Nobs | 0 |
| Mean (SD) | NA (NA) |
| Median (Q1;Q3) | NA (NA; NA) |
| Min - Max | NA, NA |
| Missing | 20 |
| - Location of administration, n/N (%) | 0/0 (NA%) |
| Missing | 20 |
| - Start date of treatment available, n/N (%) |  |
| Yes | 17/20 (85.0%) |
| No | 3/20 (15.0%) |
| - End date of treatment available, n/N (%) |  |
| No | 3/4 (75.0%) |
| Yes | 1/4 (25.0%) |
| Exemestane :  - Duration (months) |  |
| Nobs | 2 |
| Mean (SD) | 14.08 (5.04) |
| Median (Q1;Q3) | 14.1 (12.3; 15.9) |
| Min - Max | 10.5, 17.6 |
| Missing | 3 |
| - Administration frequency |  |
| Nobs | 2 |
| Mean (SD) | 4.00 (0.00) |
| Median (Q1;Q3) | 4.0 (4.0; 4.0) |
| Min - Max | 4.0, 4.0 |
| Missing | 3 |
| - Maintenance dose (cycle) |  |
| Nobs | 0 |
| Mean (SD) | NA (NA) |
| Median (Q1;Q3) | NA (NA; NA) |
| Min - Max | NA, NA |
| Missing | 5 |
| - Maintenance dose (cycle mg/kg or mg) |  |
| Nobs | 2 |
| Mean (SD) | 11.00 (12.73) |
| Median (Q1;Q3) | 11.0 (6.5; 15.5) |
| Min - Max | 2.0, 20.0 |
| Missing | 3 |
| - Route of administration, n/N (%) | 0/0 (NA%) |
| Missing | 5 |
| - Number of cycles completed |  |
| Nobs | 0 |
| Mean (SD) | NA (NA) |
| Median (Q1;Q3) | NA (NA; NA) |
| Min - Max | NA, NA |
| Missing | 5 |
| - Location of administration, n/N (%) | 0/0 (NA%) |
| Missing | 5 |
| - Start date of treatment available, n/N (%) |  |
| Yes | 4/5 (80.0%) |
| No | 1/5 (20.0%) |
| - End date of treatment available, n/N (%) |  |
| No | 1/3 (33.3%) |
| Yes | 2/3 (66.7%) |
| Letrozole :  - Duration (months) |  |
| Nobs | 8 |
| Mean (SD) | 15.65 (8.26) |
| Median (Q1;Q3) | 13.5 (9.9; 18.3) |
| Min - Max | 7.7, 30.1 |
| Missing | 32 |
| - Administration frequency |  |
| Nobs | 26 |
| Mean (SD) | 4.00 (0.00) |
| Median (Q1;Q3) | 4.0 (4.0; 4.0) |
| Min - Max | 4.0, 4.0 |
| Missing | 14 |
| - Maintenance dose (cycle) |  |
| Nobs | 1 |
| Mean (SD) | 49.00 (NA) |
| Median (Q1;Q3) | 49.0 (49.0; 49.0) |
| Min - Max | 49.0, 49.0 |
| Missing | 39 |
| - Maintenance dose (cycle mg/kg or mg) |  |
| Nobs | 9 |
| Mean (SD) | 16.22 (8.21) |
| Median (Q1;Q3) | 20.0 (19.0; 20.0) |
| Min - Max | 1.0, 24.0 |
| Missing | 31 |
| - Route of administration, n/N (%) | 0/0 (NA%) |
| Missing | 40 |
| - Number of cycles completed |  |
| Nobs | 0 |
| Mean (SD) | NA (NA) |
| Median (Q1;Q3) | NA (NA; NA) |
| Min - Max | NA, NA |
| Missing | 40 |
| - Location of administration, n/N (%) | 0/0 (NA%) |
| Missing | 40 |
| - Start date of treatment available, n/N (%) |  |
| Yes | 38/40 (95.0%) |
| No | 2/40 (5.0%) |
| - End date of treatment available, n/N (%) |  |
| No | 6/14 (42.9%) |
| Yes | 8/14 (57.1%) |
| Other :  - Duration (months) |  |
| Nobs | 2 |
| Mean (SD) | 8.30 (0.30) |
| Median (Q1;Q3) | 8.3 (8.2; 8.4) |
| Min - Max | 8.1, 8.5 |
| Missing | 0 |
| - Administration frequency |  |
| Nobs | 1 |
| Mean (SD) | 3.00 (NA) |
| Median (Q1;Q3) | 3.0 (3.0; 3.0) |
| Min - Max | 3.0, 3.0 |
| Missing | 1 |
| - Maintenance dose (cycle) |  |
| Nobs | 1 |
| Mean (SD) | 6.00 (NA) |
| Median (Q1;Q3) | 6.0 (6.0; 6.0) |
| Min - Max | 6.0, 6.0 |
| Missing | 1 |
| - Maintenance dose (cycle mg/kg or mg) |  |
| Nobs | 0 |
| Mean (SD) | NA (NA) |
| Median (Q1;Q3) | NA (NA; NA) |
| Min - Max | NA, NA |
| Missing | 2 |
| - Route of administration, n/N (%) |  |
| Subcutaneous | 1/1 (100.0%) |
| Missing | 1 |
| - Number of cycles completed |  |
| Nobs | 2 |
| Mean (SD) | 13.00 (0.00) |
| Median (Q1;Q3) | 13.0 (13.0; 13.0) |
| Min - Max | 13.0, 13.0 |
| Missing | 0 |
| - Location of administration, n/N (%) |  |
| Hospital | 1/1 (100.0%) |
| Missing | 1 |
| - Start date of treatment available, n/N (%) |  |
| Yes | 2/2 (100.0%) |
| No | 0/2 (0.0%) |
| - End date of treatment available, n/N (%) |  |
| Yes | 2/2 (100.0%) |
| Other hormonotherapy 1 :  - Duration (months) |  |
| Nobs | 0 |
| Mean (SD) | NA (NA) |
| Median (Q1;Q3) | NA (NA; NA) |
| Min - Max | NA, NA |
| Missing | 3 |
| - Administration frequency |  |
| Nobs | 1 |
| Mean (SD) | 4.00 (NA) |
| Median (Q1;Q3) | 4.0 (4.0; 4.0) |
| Min - Max | 4.0, 4.0 |
| Missing | 2 |
| - Maintenance dose (cycle) |  |
| Nobs | 0 |
| Mean (SD) | NA (NA) |
| Median (Q1;Q3) | NA (NA; NA) |
| Min - Max | NA, NA |
| Missing | 3 |
| - Maintenance dose (cycle mg/kg or mg) |  |
| Nobs | 1 |
| Mean (SD) | 20.00 (NA) |
| Median (Q1;Q3) | 20.0 (20.0; 20.0) |
| Min - Max | 20.0, 20.0 |
| Missing | 2 |
| - Route of administration, n/N (%) | 0/0 (NA%) |
| Missing | 3 |
| - Number of cycles completed |  |
| Nobs | 0 |
| Mean (SD) | NA (NA) |
| Median (Q1;Q3) | NA (NA; NA) |
| Min - Max | NA, NA |
| Missing | 3 |
| - Location of administration, n/N (%) | 0/0 (NA%) |
| Missing | 3 |
| - Start date of treatment available, n/N (%) |  |
| Yes | 2/3 (66.7%) |
| No | 1/3 (33.3%) |
| - End date of treatment available, n/N (%) |  |
| No | 3/3 (100.0%) |
| Tamoxifene :  - Duration (months) |  |
| Nobs | 24 |
| Mean (SD) | 23.06 (13.74) |
| Median (Q1;Q3) | 22.4 (17.8; 33.4) |
| Min - Max | -23.8, 42.3 |
| Missing | 57 |
| - Administration frequency |  |
| Nobs | 56 |
| Mean (SD) | 4.00 (0.00) |
| Median (Q1;Q3) | 4.0 (4.0; 4.0) |
| Min - Max | 4.0, 4.0 |
| Missing | 25 |
| - Maintenance dose (cycle) |  |
| Nobs | 11 |
| Mean (SD) | 18.55 (4.82) |
| Median (Q1;Q3) | 20.0 (20.0; 20.0) |
| Min - Max | 4.0, 20.0 |
| Missing | 70 |
| - Maintenance dose (cycle mg/kg or mg) |  |
| Nobs | 35 |
| Mean (SD) | 19.00 (4.60) |
| Median (Q1;Q3) | 20.0 (20.0; 20.0) |
| Min - Max | 1.0, 25.0 |
| Missing | 46 |
| - Route of administration, n/N (%) | 0/0 (NA%) |
| Missing | 81 |
| - Number of cycles completed |  |
| Nobs | 0 |
| Mean (SD) | NA (NA) |
| Median (Q1;Q3) | NA (NA; NA) |
| Min - Max | NA, NA |
| Missing | 81 |
| - Location of administration, n/N (%) | 0/0 (NA%) |
| Missing | 81 |
| - Start date of treatment available, n/N (%) |  |
| Yes | 77/81 (95.1%) |
| No | 4/81 (4.9%) |
| - End date of treatment available, n/N (%) |  |
| No | 19/43 (44.2%) |
| Yes | 24/43 (55.8%) |
| Trastuzumab (Herceptin) :  - Duration (months) |  |
| Nobs | 297 |
| Mean (SD) | 8.14 (2.98) |
| Median (Q1;Q3) | 8.8 (8.0; 9.2) |
| Min - Max | -32.3, 12.6 |
| Missing | 7 |
| - Administration frequency |  |
| Nobs | 257 |
| Mean (SD) | 3.02 (0.15) |
| Median (Q1;Q3) | 3.0 (3.0; 3.0) |
| Min - Max | 3.0, 4.0 |
| Missing | 47 |
| - Maintenance dose (cycle) |  |
| Nobs | 264 |
| Mean (SD) | 7.76 (23.79) |
| Median (Q1;Q3) | 6.0 (6.0; 6.0) |
| Min - Max | 6.0, 391.0 |
| Missing | 40 |
| - Maintenance dose (cycle mg/kg or mg) |  |
| Nobs | 30 |
| Mean (SD) | 572.00 (81.20) |
| Median (Q1;Q3) | 600.0 (600.0; 600.0) |
| Min - Max | 326.0, 600.0 |
| Missing | 274 |
| - Route of administration, n/N (%) |  |
| Both | 40/261 (15.3%) |
| Intravenous | 162/261 (62.1%) |
| Subcutaneous | 59/261 (22.6%) |
| Missing | 43 |
| - Number of cycles completed |  |
| Nobs | 294 |
| Mean (SD) | 13.30 (2.55) |
| Median (Q1;Q3) | 14.0 (12.0; 14.0) |
| Min - Max | 3.0, 19.0 |
| Missing | 10 |
| - Location of administration, n/N (%) |  |
| Home | 6/283 (2.1%) |
| Hospital | 277/283 (97.9%) |
| Missing | 21 |
| - Start date of treatment available, n/N (%) |  |
| Yes | 303/304 (99.7%) |
| No | 1/304 (0.3%) |
| - End date of treatment available, n/N (%) |  |
| No | 6/303 (2.0%) |
| Yes | 297/303 (98.0%) |
| Duration of each adjuvant (months) = (End date of treatment – Start date of treatment + 1) / (365.25/12) | |

## Table 4.3 Time between surgery and adjuvant treatment - Among subjects with at least one adjuvant treatments - Full Analysis Set Population

| Characteristic | All (N = 305) |
| --- | --- |
| Time from surgery to adjuvant treatment initiation of Herceptin (days) |  |
| Nobs | 301 |
| Mean (SD) | 84.10 (1,768.07) |
| Median (Q1;Q3) | 70.0 (-1,039.0; 1,137.0) |
| Min - Max | -4,569.0, 8,608.0 |
| Missing | 4 |
| Time from surgery to adjuvant treatment initiation of Herceptin (days) = (Date of adjuvant treatment initiation of Herceptin - Surgery date) | |

## Table 4.4 Summary of adjuvant treatments by pCR status - Among subjects with at least one adjuvant treatments - Full Analysis Set Population

| Characteristic | pCR (N = 124) | No pCR (N = 181) |
| --- | --- | --- |
| At least one Adjuvant Treatment* |  |  |
| Trastuzumab (Herceptin) | 124 (100%) | 180 (99.4%) |
| Tamoxifene | 30 (24.2%) | 51 (28.2%) |
| Letrozole | 16 (12.9%) | 24 (13.3%) |
| Anastrozole | 9 (7.3%) | 11 (6.1%) |
| Exemestane | 3 (2.4%) | 2 (1.1%) |
| Other hormonotherapy 1 | 1 (0.8%) | 2 (1.1%) |
| Other | 1 (0.8%) | 1 (0.6%) |

## Table 4.5 Summary of adjuvant treatments by adjuvant treatment by pCR status - Among subjects with at least one adjuvant treatments - Full Analysis Set Population

| Characteristic | pCR (N = 124) | No pCR (N = 181) |
| --- | --- | --- |
| Anastrozole :  - Duration (months) |  |  |
| Nobs | 0 | 1 |
| Mean (SD) | NA (NA) | 15.80 (NA) |
| Median (Q1;Q3) | NA (NA; NA) | 15.8 (15.8; 15.8) |
| Min - Max | NA, NA | 15.8, 15.8 |
| Missing | 9 | 10 |
| - Administration frequency |  |  |
| Nobs | 7 | 9 |
| Mean (SD) | 4.00 (0.00) | 4.00 (0.00) |
| Median (Q1;Q3) | 4.0 (4.0; 4.0) | 4.0 (4.0; 4.0) |
| Min - Max | 4.0, 4.0 | 4.0, 4.0 |
| Missing | 2 | 2 |
| - Maintenance dose (cycle) |  |  |
| Nobs | 0 | 0 |
| Mean (SD) | NA (NA) | NA (NA) |
| Median (Q1;Q3) | NA (NA; NA) | NA (NA; NA) |
| Min - Max | NA, NA | NA, NA |
| Missing | 9 | 11 |
| - Maintenance dose (cycle mg/kg or mg) |  |  |
| Nobs | 2 | 4 |
| Mean (SD) | 11.50 (12.02) | 11.25 (10.11) |
| Median (Q1;Q3) | 11.5 (7.2; 15.8) | 11.5 (2.8; 20.0) |
| Min - Max | 3.0, 20.0 | 2.0, 20.0 |
| Missing | 7 | 7 |
| - Route of administration, n/N (%) | 0/0 (NA%) | 0/0 (NA%) |
| Missing | 9 | 11 |
| - Number of cycles completed |  |  |
| Nobs | 0 | 0 |
| Mean (SD) | NA (NA) | NA (NA) |
| Median (Q1;Q3) | NA (NA; NA) | NA (NA; NA) |
| Min - Max | NA, NA | NA, NA |
| Missing | 9 | 11 |
| - Location of administration, n/N (%) | 0/0 (NA%) | 0/0 (NA%) |
| Missing | 9 | 11 |
| Exemestane :  - Duration (months) |  |  |
| Nobs | 2 | 0 |
| Mean (SD) | 14.08 (5.04) | NA (NA) |
| Median (Q1;Q3) | 14.1 (12.3; 15.9) | NA (NA; NA) |
| Min - Max | 10.5, 17.6 | NA, NA |
| Missing | 1 | 2 |
| - Administration frequency |  |  |
| Nobs | 1 | 1 |
| Mean (SD) | 4.00 (NA) | 4.00 (NA) |
| Median (Q1;Q3) | 4.0 (4.0; 4.0) | 4.0 (4.0; 4.0) |
| Min - Max | 4.0, 4.0 | 4.0, 4.0 |
| Missing | 2 | 1 |
| - Maintenance dose (cycle) |  |  |
| Nobs | 0 | 0 |
| Mean (SD) | NA (NA) | NA (NA) |
| Median (Q1;Q3) | NA (NA; NA) | NA (NA; NA) |
| Min - Max | NA, NA | NA, NA |
| Missing | 3 | 2 |
| - Maintenance dose (cycle mg/kg or mg) |  |  |
| Nobs | 1 | 1 |
| Mean (SD) | 20.00 (NA) | 2.00 (NA) |
| Median (Q1;Q3) | 20.0 (20.0; 20.0) | 2.0 (2.0; 2.0) |
| Min - Max | 20.0, 20.0 | 2.0, 2.0 |
| Missing | 2 | 1 |
| - Route of administration, n/N (%) | 0/0 (NA%) | 0/0 (NA%) |
| Missing | 3 | 2 |
| - Number of cycles completed |  |  |
| Nobs | 0 | 0 |
| Mean (SD) | NA (NA) | NA (NA) |
| Median (Q1;Q3) | NA (NA; NA) | NA (NA; NA) |
| Min - Max | NA, NA | NA, NA |
| Missing | 3 | 2 |
| - Location of administration, n/N (%) | 0/0 (NA%) | 0/0 (NA%) |
| Missing | 3 | 2 |
| Letrozole :  - Duration (months) |  |  |
| Nobs | 1 | 7 |
| Mean (SD) | 8.21 (NA) | 16.71 (8.31) |
| Median (Q1;Q3) | 8.2 (8.2; 8.2) | 15.3 (11.0; 20.9) |
| Min - Max | 8.2, 8.2 | 7.7, 30.1 |
| Missing | 15 | 17 |
| - Administration frequency |  |  |
| Nobs | 9 | 17 |
| Mean (SD) | 4.00 (0.00) | 4.00 (0.00) |
| Median (Q1;Q3) | 4.0 (4.0; 4.0) | 4.0 (4.0; 4.0) |
| Min - Max | 4.0, 4.0 | 4.0, 4.0 |
| Missing | 7 | 7 |
| - Maintenance dose (cycle) |  |  |
| Nobs | 1 | 0 |
| Mean (SD) | 49.00 (NA) | NA (NA) |
| Median (Q1;Q3) | 49.0 (49.0; 49.0) | NA (NA; NA) |
| Min - Max | 49.0, 49.0 | NA, NA |
| Missing | 15 | 24 |
| - Maintenance dose (cycle mg/kg or mg) |  |  |
| Nobs | 5 | 4 |
| Mean (SD) | 15.80 (8.29) | 16.75 (9.36) |
| Median (Q1;Q3) | 19.0 (19.0; 20.0) | 20.0 (15.8; 21.0) |
| Min - Max | 1.0, 20.0 | 3.0, 24.0 |
| Missing | 11 | 20 |
| - Route of administration, n/N (%) | 0/0 (NA%) | 0/0 (NA%) |
| Missing | 16 | 24 |
| - Number of cycles completed |  |  |
| Nobs | 0 | 0 |
| Mean (SD) | NA (NA) | NA (NA) |
| Median (Q1;Q3) | NA (NA; NA) | NA (NA; NA) |
| Min - Max | NA, NA | NA, NA |
| Missing | 16 | 24 |
| - Location of administration, n/N (%) | 0/0 (NA%) | 0/0 (NA%) |
| Missing | 16 | 24 |
| Other :  - Duration (months) |  |  |
| Nobs | 1 | 1 |
| Mean (SD) | 8.08 (NA) | 8.51 (NA) |
| Median (Q1;Q3) | 8.1 (8.1; 8.1) | 8.5 (8.5; 8.5) |
| Min - Max | 8.1, 8.1 | 8.5, 8.5 |
| Missing | 0 | 0 |
| - Administration frequency |  |  |
| Nobs | 0 | 1 |
| Mean (SD) | NA (NA) | 3.00 (NA) |
| Median (Q1;Q3) | NA (NA; NA) | 3.0 (3.0; 3.0) |
| Min - Max | NA, NA | 3.0, 3.0 |
| Missing | 1 | 0 |
| - Maintenance dose (cycle) |  |  |
| Nobs | 0 | 1 |
| Mean (SD) | NA (NA) | 6.00 (NA) |
| Median (Q1;Q3) | NA (NA; NA) | 6.0 (6.0; 6.0) |
| Min - Max | NA, NA | 6.0, 6.0 |
| Missing | 1 | 0 |
| - Maintenance dose (cycle mg/kg or mg) |  |  |
| Nobs | 0 | 0 |
| Mean (SD) | NA (NA) | NA (NA) |
| Median (Q1;Q3) | NA (NA; NA) | NA (NA; NA) |
| Min - Max | NA, NA | NA, NA |
| Missing | 1 | 1 |
| - Route of administration, n/N (%) |  |  |
| Subcutaneous | 1/1 (100.0%) | 0/0 (NA%) |
| Missing | 0 | 1 |
| - Number of cycles completed |  |  |
| Nobs | 1 | 1 |
| Mean (SD) | 13.00 (NA) | 13.00 (NA) |
| Median (Q1;Q3) | 13.0 (13.0; 13.0) | 13.0 (13.0; 13.0) |
| Min - Max | 13.0, 13.0 | 13.0, 13.0 |
| Missing | 0 | 0 |
| - Location of administration, n/N (%) |  |  |
| Hospital | 0/0 (NA%) | 1/1 (100.0%) |
| Missing | 1 | 0 |
| Other hormonotherapy 1 :  - Duration (months) |  |  |
| Nobs | 0 | 0 |
| Mean (SD) | NA (NA) | NA (NA) |
| Median (Q1;Q3) | NA (NA; NA) | NA (NA; NA) |
| Min - Max | NA, NA | NA, NA |
| Missing | 1 | 2 |
| - Administration frequency |  |  |
| Nobs | 1 | 0 |
| Mean (SD) | 4.00 (NA) | NA (NA) |
| Median (Q1;Q3) | 4.0 (4.0; 4.0) | NA (NA; NA) |
| Min - Max | 4.0, 4.0 | NA, NA |
| Missing | 0 | 2 |
| - Maintenance dose (cycle) |  |  |
| Nobs | 0 | 0 |
| Mean (SD) | NA (NA) | NA (NA) |
| Median (Q1;Q3) | NA (NA; NA) | NA (NA; NA) |
| Min - Max | NA, NA | NA, NA |
| Missing | 1 | 2 |
| - Maintenance dose (cycle mg/kg or mg) |  |  |
| Nobs | 1 | 0 |
| Mean (SD) | 20.00 (NA) | NA (NA) |
| Median (Q1;Q3) | 20.0 (20.0; 20.0) | NA (NA; NA) |
| Min - Max | 20.0, 20.0 | NA, NA |
| Missing | 0 | 2 |
| - Route of administration, n/N (%) | 0/0 (NA%) | 0/0 (NA%) |
| Missing | 1 | 2 |
| - Number of cycles completed |  |  |
| Nobs | 0 | 0 |
| Mean (SD) | NA (NA) | NA (NA) |
| Median (Q1;Q3) | NA (NA; NA) | NA (NA; NA) |
| Min - Max | NA, NA | NA, NA |
| Missing | 1 | 2 |
| - Location of administration, n/N (%) | 0/0 (NA%) | 0/0 (NA%) |
| Missing | 1 | 2 |
| Tamoxifene :  - Duration (months) |  |  |
| Nobs | 9 | 15 |
| Mean (SD) | 18.62 (18.96) | 25.73 (9.20) |
| Median (Q1;Q3) | 20.8 (11.5; 31.6) | 22.7 (19.2; 33.8) |
| Min - Max | -23.8, 38.1 | 10.5, 42.3 |
| Missing | 21 | 36 |
| - Administration frequency |  |  |
| Nobs | 21 | 35 |
| Mean (SD) | 4.00 (0.00) | 4.00 (0.00) |
| Median (Q1;Q3) | 4.0 (4.0; 4.0) | 4.0 (4.0; 4.0) |
| Min - Max | 4.0, 4.0 | 4.0, 4.0 |
| Missing | 9 | 16 |
| - Maintenance dose (cycle) |  |  |
| Nobs | 4 | 7 |
| Mean (SD) | 20.00 (0.00) | 17.71 (6.05) |
| Median (Q1;Q3) | 20.0 (20.0; 20.0) | 20.0 (20.0; 20.0) |
| Min - Max | 20.0, 20.0 | 4.0, 20.0 |
| Missing | 26 | 44 |
| - Maintenance dose (cycle mg/kg or mg) |  |  |
| Nobs | 12 | 23 |
| Mean (SD) | 18.25 (5.46) | 19.39 (4.16) |
| Median (Q1;Q3) | 20.0 (20.0; 20.0) | 20.0 (20.0; 20.0) |
| Min - Max | 1.0, 20.0 | 2.0, 25.0 |
| Missing | 18 | 28 |
| - Route of administration, n/N (%) | 0/0 (NA%) | 0/0 (NA%) |
| Missing | 30 | 51 |
| - Number of cycles completed |  |  |
| Nobs | 0 | 0 |
| Mean (SD) | NA (NA) | NA (NA) |
| Median (Q1;Q3) | NA (NA; NA) | NA (NA; NA) |
| Min - Max | NA, NA | NA, NA |
| Missing | 30 | 51 |
| - Location of administration, n/N (%) | 0/0 (NA%) | 0/0 (NA%) |
| Missing | 30 | 51 |
| Trastuzumab (Herceptin) :  - Duration (months) |  |  |
| Nobs | 122 | 175 |
| Mean (SD) | 8.42 (1.72) | 7.94 (3.59) |
| Median (Q1;Q3) | 9.0 (8.1; 9.3) | 8.7 (7.6; 9.2) |
| Min - Max | 1.4, 11.6 | -32.3, 12.6 |
| Missing | 2 | 5 |
| - Administration frequency |  |  |
| Nobs | 100 | 157 |
| Mean (SD) | 3.04 (0.20) | 3.01 (0.11) |
| Median (Q1;Q3) | 3.0 (3.0; 3.0) | 3.0 (3.0; 3.0) |
| Min - Max | 3.0, 4.0 | 3.0, 4.0 |
| Missing | 24 | 23 |
| - Maintenance dose (cycle) |  |  |
| Nobs | 106 | 158 |
| Mean (SD) | 6.62 (3.62) | 8.53 (30.63) |
| Median (Q1;Q3) | 6.0 (6.0; 6.0) | 6.0 (6.0; 6.0) |
| Min - Max | 6.0, 40.0 | 6.0, 391.0 |
| Missing | 18 | 22 |
| - Maintenance dose (cycle mg/kg or mg) |  |  |
| Nobs | 14 | 16 |
| Mean (SD) | 578.79 (72.95) | 566.06 (89.75) |
| Median (Q1;Q3) | 600.0 (600.0; 600.0) | 600.0 (600.0; 600.0) |
| Min - Max | 326.0, 600.0 | 328.0, 600.0 |
| Missing | 110 | 164 |
| - Route of administration, n/N (%) |  |  |
| Both | 15/106 (14.2%) | 25/155 (16.1%) |
| Intravenous | 63/106 (59.4%) | 99/155 (63.9%) |
| Subcutaneous | 28/106 (26.4%) | 31/155 (20.0%) |
| Missing | 18 | 25 |
| - Number of cycles completed |  |  |
| Nobs | 121 | 173 |
| Mean (SD) | 13.26 (2.68) | 13.32 (2.46) |
| Median (Q1;Q3) | 14.0 (13.0; 14.0) | 14.0 (12.0; 14.0) |
| Min - Max | 3.0, 18.0 | 3.0, 19.0 |
| Missing | 3 | 7 |
| - Location of administration, n/N (%) |  |  |
| Home | 3/116 (2.6%) | 3/167 (1.8%) |
| Hospital | 113/116 (97.4%) | 164/167 (98.2%) |
| Missing | 8 | 13 |
| Duration of each adjuvant (months) = (End date of treatment – Start date of treatment + 1) / (365.25/12) | | |

## Table 4.6 Time between surgery and adjuvant treatment by pCR status - Among subjects with at least one adjuvant treatments - Full Analysis Set Population

| Characteristic | pCR (N = 124) | No pCR (N = 181) |
| --- | --- | --- |
| Time from surgery to adjuvant treatment initiation of Herceptin (days) |  |  |
| Nobs | 121 | 180 |
| Mean (SD) | -33.50 (1,675.56) | 163.16 (1,827.97) |
| Median (Q1;Q3) | -145.0 (-1,039.0; 895.0) | 146.0 (-1,036.0; 1,252.5) |
| Min - Max | -4,569.0, 4,777.0 | -3,955.0, 8,608.0 |
| Missing | 3 | 1 |
| Time from surgery to adjuvant treatment initiation of Herceptin (days) = (Date of adjuvant treatment initiation of Herceptin - Surgery date) | | |

# 5 Efficacy Analyses

## 5.1 Time to event analyses

### Table 5.1.1 Summary of time from herceptin adjuvant treatment to PFS, overall and by pCR result - Kaplan-Meier estimation - Among subjects with herceptin adjuvant treatment start date available - Full Analysis Set Population

| PFS | N | Number of event | 10% Percentile (95% CI) |
| --- | --- | --- | --- |
| Overall | 297 | 37 | 4.6 (0.65, 8.3) |
| pCR results* |  |  |  |
| pCR | 122 | 9 | 10 (1.3, 12) |
| No pCR | 175 | 28 | 3.5 (-2.3, 7.0) |
| * pCR results = pCR if ypT0/Tis ypN0 is ticked Yes OR, Grade 1 or Grade 2 are ticked for Classification Chevallier OR, TA and NA are ticked for Classification Sataloff OR, RCB0 is ticked for Classification RCB | | | |
| Patients who did not experience event were censored at their last consultation date. If this date is missing, they were censored at the last adjuvant treatment date | | | |

### Table 5.1.2 Survival probabilities of time from herceptin adjuvant treatment to PFS, overall and by pCR result - Kaplan-Meier estimation - Among subjects with herceptin adjuvant treatment start date available - Full Analysis Set Population

| PFS | N | At 1 year (95% CI) | At 2 years (95% CI) | At 3 years (95% CI) | At 4 years (95% CI) |
| --- | --- | --- | --- | --- | --- |
| Overall | 297 | 93.6 (90.0, 95.9) | 92.6 (88.7, 95.2) | 92.6 (88.7, 95.2) | 90.6 (85.9, 93.7) |
| pCR results* |  |  |  |  |  |
| pCR | 122 | 96.6 (91.2, 98.7) | 95.5 (89.3, 98.1) | 95.5 (89.3, 98.1) | 93.8 (86.3, 97.3) |
| No pCR | 175 | 91.5 (86.0, 94.9) | 90.6 (84.9, 94.3) | 90.6 (84.9, 94.3) | 88.3 (81.6, 92.7) |
| * pCR results = pCR if ypT0/Tis ypN0 is ticked Yes OR, Grade 1 or Grade 2 are ticked for Classification Chevallier OR, TA and NA are ticked for Classification Sataloff OR, RCB0 is ticked for Classification RCB | | | | | |
| Patients who did not experience event were censored at their last consultation date. If this date is missing, they were censored at the last adjuvant treatment date | | | | | |

### Table 5.1.3 Summary of time from herceptin adjuvant treatment to PFS - Kaplan-Meier curve - Among subjects with herceptin adjuvant treatment start date available - Full Analysis Set Population


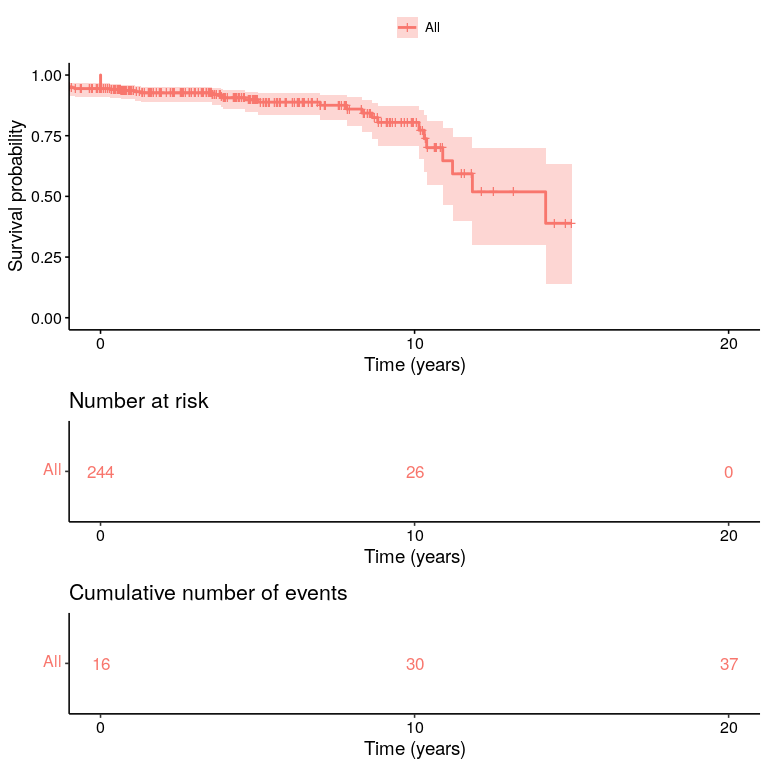


### Table 5.1.4 Summary of time from herceptin adjuvant treatment to PFS by pCR result - Kaplan-Meier curve - Among subjects with herceptin adjuvant treatment start date available - Full Analysis Set Population


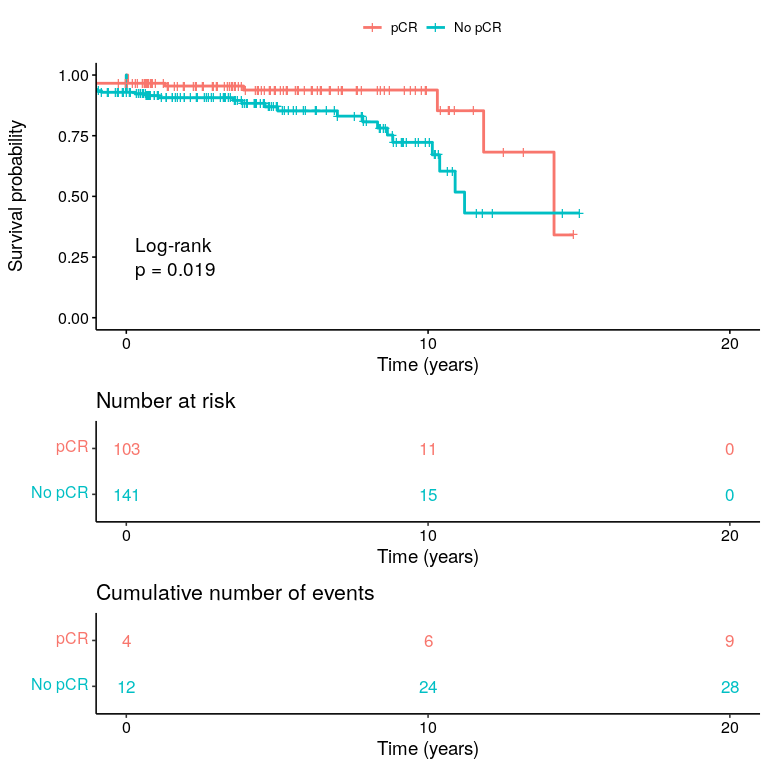


# 6 Exploratory Analyses

## 6.1 Predictive factors for PFS

### Table 6.1.1 PFS - Univariate Cox proportional hazard analysis - Among subjects with herceptin adjuvant treatment start date available - Full Analysis Set Population

|  | Descriptive statistics | | HR and 95% CI | | |
| --- | --- | --- | --- | --- | --- |
| Characteristic | Event, N = 43 | No event, N = 260 | HR^1^ | 95% CI^1^ | p-value |
| Age at adjuvant treatment initiation of Herceptin (years) |  |  | 0.99 | 0.96, 1.01 | 0.325 |
| N | 43 | 260 |  |  |  |
| Mean (SD) | 50.65 (13.01) | 52.49 (11.61) |  |  |  |
| Median (25%; 75%) | 54.0 (38.0; 59.0) | 52.0 (44.0; 61.0) |  |  |  |
| Range | 29, 76 | 29, 77 |  |  |  |
| Missing | 0 | 0 |  |  |  |
| Age group (years) |  |  |  |  | 0.047 |
| <40 | 12/53 (22.6%) | 41/53 (77.4%) | — | — |  |
| [40 - 49] | 5/75 (6.7%) | 70/75 (93.3%) | 0.27 | 0.09, 0.83 |  |
| [50 - 59] | 16/91 (17.6%) | 75/91 (82.4%) | 1.14 | 0.52, 2.51 |  |
| [60 - 69] | 8/60 (13.3%) | 52/60 (86.7%) | 0.40 | 0.14, 1.14 |  |
| >=70 | 2/24 (8.3%) | 22/24 (91.7%) | 0.51 | 0.11, 2.32 |  |
| Missing | 0 | 0 |  |  |  |
| BMI (kg/m2) |  |  |  |  | 0.940 |
| <25 | 19/141 (13.5%) | 122/141 (86.5%) | — | — |  |
| [25 - 30[ | 16/112 (14.3%) | 96/112 (85.7%) | 0.90 | 0.43, 1.86 |  |
| >=30 | 7/48 (14.6%) | 41/48 (85.4%) | 1.04 | 0.41, 2.66 |  |
| Missing | 1 | 1 |  |  |  |
| T classification |  |  |  |  | 0.038 |
| T0-3 | 31/267 (11.6%) | 236/267 (88.4%) | — | — |  |
| T>3 | 12/34 (35.3%) | 22/34 (64.7%) | 2.24 | 1.04, 4.79 |  |
| Missing | 0 | 2 |  |  |  |
| N classification |  |  |  |  | 0.496 |
| N0 | 17/108 (15.7%) | 91/108 (84.3%) | — | — |  |
| N1 | 22/142 (15.5%) | 120/142 (84.5%) | 1.30 | 0.65, 2.59 |  |
| N2&N3 | 3/24 (12.5%) | 21/24 (87.5%) | 0.59 | 0.13, 2.60 |  |
| Missing | 1 | 28 |  |  |  |
| SBR Grade |  |  |  |  | 0.207 |
| SBR I & II | 18/143 (12.6%) | 125/143 (87.4%) | — | — |  |
| SBR III | 24/152 (15.8%) | 128/152 (84.2%) | 1.53 | 0.79, 2.95 |  |
| Missing | 1 | 7 |  |  |  |
| Presence of vascular emboli |  |  |  |  | 0.005 |
| Yes | 5/15 (33.3%) | 10/15 (66.7%) | — | — |  |
| No | 24/213 (11.3%) | 189/213 (88.7%) | 0.24 | 0.09, 0.64 |  |
| Missing | 14 | 61 |  |  |  |
| Hormonal receptors status |  |  |  |  | 0.054 |
| ER and/or PR + | 23/181 (12.7%) | 158/181 (87.3%) | — | — |  |
| ER and PR - | 20/121 (16.5%) | 101/121 (83.5%) | 1.93 | 0.99, 3.77 |  |
| Missing | 0 | 1 |  |  |  |
| pCR results* |  |  |  |  | 0.023 |
| pCR | 10/123 (8.1%) | 113/123 (91.9%) | — | — |  |
| No pCR | 33/180 (18.3%) | 147/180 (81.7%) | 2.39 | 1.13, 5.09 |  |
| Missing | 0 | 0 |  |  |  |
| * pCR results = pCR if ypT0/Tis ypN0 is ticked Yes OR, Grade 1 or Grade 2 are ticked for Classification Chevallier OR, TA and NA are ticked for Classification Sataloff OR, RCB0 is ticked for Classification RCB | | | | | |
| Univariate analysis has been done using a cox model. P-value is based on a global wald test from Cox model | | | | | |
| ^1^HR = Hazard Ratio, CI = Confidence Interval | | | | | |

### Table 6.1.2 PFS - Multivariate Cox proportional hazard analysis - Among subjects with herceptin adjuvant treatment start date available - Full Analysis Set Population

|  | Descriptive statistics | | HR and 95% CI | | |
| --- | --- | --- | --- | --- | --- |
| Characteristic | Event, N = 43 | No event, N = 260 | HR^1^ | 95% CI^1^ | p-value |
| Presence of vascular emboli |  |  |  |  | <0.001 |
| Yes | 5/15 (33.3%) | 10/15 (66.7%) | — | — |  |
| No | 24/213 (11.3%) | 189/213 (88.7%) | 0.15 | 0.05, 0.45 |  |
| Missing | 14 | 61 |  |  |  |
| Age group (years) |  |  |  |  | 0.009 |
| <40 | 12/53 (22.6%) | 41/53 (77.4%) | — | — |  |
| [40 - 49] | 5/75 (6.7%) | 70/75 (93.3%) | 0.06 | 0.01, 0.50 |  |
| [50 - 59] | 16/91 (17.6%) | 75/91 (82.4%) | 0.90 | 0.34, 2.41 |  |
| [60 - 69] | 8/60 (13.3%) | 52/60 (86.7%) | 0.19 | 0.05, 0.67 |  |
| >=70 | 2/24 (8.3%) | 22/24 (91.7%) | 0.40 | 0.08, 2.09 |  |
| Missing | 0 | 0 |  |  |  |
| pCR results* |  |  |  |  | 0.017 |
| pCR | 10/123 (8.1%) | 113/123 (91.9%) | — | — |  |
| No pCR | 33/180 (18.3%) | 147/180 (81.7%) | 3.25 | 1.24, 8.55 |  |
| Missing | 0 | 0 |  |  |  |
| T classification |  |  |  |  | 0.005 |
| T0-3 | 31/267 (11.6%) | 236/267 (88.4%) | — | — |  |
| T>3 | 12/34 (35.3%) | 22/34 (64.7%) | 4.72 | 1.62, 13.8 |  |
| Missing | 0 | 2 |  |  |  |
| * pCR results = pCR if ypT0/Tis ypN0 is ticked Yes OR, Grade 1 or Grade 2 are ticked for Classification Chevallier OR, TA and NA are ticked for Classification Sataloff OR, RCB0 is ticked for Classification RCB | | | | | |
| Multivariate analysis has been done using a cox model. For covariate with 2 modalities, p-value is based on a global wald test from Cox model, otherwise it is the likelihood ratio test global p-value. The model has been constructed using a stepwise selection of covariates with 0.15 as entry threshold and 0.15 as the retention threshold. | | | | | |
| ^1^HR = Hazard Ratio, CI = Confidence Interval | | | | | |

## 6.2 Predictive factors for pCR result

### Table 6.2.1 pCR result - Univariate analysis - Full Analysis Set Population

|  | Descriptive statistics | | OR and 95% CI | | |
| --- | --- | --- | --- | --- | --- |
| Characteristic | pCR, N = 127 | No pCR, N = 188 | OR^1^ | 95% CI^1^ | p-value |
| Age at adjuvant treatment initiation of Herceptin (years) |  |  | 1.00 | 0.98, 1.02 | 0.997 |
| N | 123 | 180 |  |  |  |
| Mean (SD) | 52.23 (12.26) | 52.23 (11.54) |  |  |  |
| Median (25%; 75%) | 52.0 (44.0; 61.5) | 53.0 (43.0; 60.0) |  |  |  |
| Range | 29, 77 | 29, 76 |  |  |  |
| Missing | 4 | 8 |  |  |  |
| Age group (years) |  |  |  |  | 0.701 |
| <40 | 23/53 (43.4%) | 30/53 (56.6%) | — | — |  |
| [40 - 49] | 30/75 (40.0%) | 45/75 (60.0%) | 0.87 | 0.43, 1.78 |  |
| [50 - 59] | 36/91 (39.6%) | 55/91 (60.4%) | 0.85 | 0.43, 1.70 |  |
| [60 - 69] | 21/60 (35.0%) | 39/60 (65.0%) | 0.70 | 0.33, 1.50 |  |
| >=70 | 13/24 (54.2%) | 11/24 (45.8%) | 1.54 | 0.59, 4.13 |  |
| Missing | 4 | 8 |  |  |  |
| BMI (kg/m2) |  |  |  |  | 0.946 |
| <25 | 63/146 (43.2%) | 83/146 (56.8%) | — | — |  |
| [25 - 30[ | 50/117 (42.7%) | 67/117 (57.3%) | 0.98 | 0.60, 1.61 |  |
| >=30 | 14/50 (28.0%) | 36/50 (72.0%) | 0.51 | 0.25, 1.01 |  |
| Missing | 0 | 2 |  |  |  |
| T classification |  |  |  |  | 0.526 |
| T0-3 | 112/276 (40.6%) | 164/276 (59.4%) | — | — |  |
| T>3 | 13/37 (35.1%) | 24/37 (64.9%) | 0.79 | 0.38, 1.60 |  |
| Missing | 2 | 0 |  |  |  |
| N classification |  |  |  |  | 0.749 |
| N0 | 42/112 (37.5%) | 70/112 (62.5%) | — | — |  |
| N1 | 58/147 (39.5%) | 89/147 (60.5%) | 1.09 | 0.66, 1.81 |  |
| N2&N3 | 14/25 (56.0%) | 11/25 (44.0%) | 2.12 | 0.89, 5.20 |  |
| Missing | 13 | 18 |  |  |  |
| SBR Grade |  |  |  |  | 0.912 |
| SBR I & II | 58/144 (40.3%) | 86/144 (59.7%) | — | — |  |
| SBR III | 63/154 (40.9%) | 91/154 (59.1%) | 1.03 | 0.65, 1.63 |  |
| Missing | 6 | 11 |  |  |  |
| Presence of vascular emboli |  |  |  |  | 0.797 |
| Yes | 7/15 (46.7%) | 8/15 (53.3%) | — | — |  |
| No | 93/215 (43.3%) | 122/215 (56.7%) | 0.87 | 0.30, 2.57 |  |
| Missing | 27 | 58 |  |  |  |
| Hormonal receptors status |  |  |  |  | 0.668 |
| ER and/or PR + | 72/183 (39.3%) | 111/183 (60.7%) | — | — |  |
| ER and PR - | 51/122 (41.8%) | 71/122 (58.2%) | 1.11 | 0.69, 1.76 |  |
| Missing | 4 | 6 |  |  |  |
| Univariate analysis has been done using a logistic model. P-value is based on a global wald test from logistic model | | | | | |
| ^1^OR = Odds Ratio, CI = Confidence Interval | | | | | |

### Table 6.2.2 pCR result - Multivariate analysis - Full Analysis Set Population

No covariate with p-value<0.15

## 6.3 Predictive factors for PFS and pCR result

### Table 6.3.1 Correlation matrix - Full Analysis Set Population

| Variables | Age (years) | Age group (years) | BMI (kg/m2) | T classification | N classification | SBR Grade | Presence of vascular emboli | Hormonal receptors status | pCR results |
| --- | --- | --- | --- | --- | --- | --- | --- | --- | --- |
| Age (years) | ND |  |  |  |  |  |  |  |  |
| Age group (years) | ND | ND |  |  |  |  |  |  |  |
| BMI (kg/m2) | 0 | 5e-04 | ND |  |  |  |  |  |  |
| T classification | 0.0896 | 0.0205 | 0.0142 | ND |  |  |  |  |  |
| N classification | 3e-04 | 0.0185 | 0.4888 | 0.0455 | ND |  |  |  |  |
| SBR Grade | 0.8627 | 0.9471 | 0.0356 | 1 | 0.0819 | ND |  |  |  |
| Presence of vascular emboli | 0.7479 | 0.9075 | 0.8201 | 0.3723 | 0.1499 | 0.3014 | ND |  |  |
| Hormonal receptors status | 0.0053 | 0.0413 | 0.092 | 0.1132 | 0.0021 | 0.011 | 0.7476 | ND |  |
| pCR results | 0.9967 | 0.5857 | 0.1418 | 0.6482 | 0.2264 | 1 | 1 | 0.7568 | ND |
| * pCR results = pCR if ypT0/Tis ypN0 is ticked Yes OR, Grade 1 or Grade 2 are ticked for Classification Chevallier OR, TA and NA are ticked for Classification Sataloff OR, RCB0 is ticked for Classification RCB | | | | | | | | | |
| ND: Not Done | | | | | | | | | |
| Between quantitative and qualitative variables: Anova have been used: the p-value displayed is the p-value of the Type 3 test of fixed effects. P-value is displayed in the above table. | | | | | | | | | |
| Between qualitative variables: Chi² test has been used when all expected counts are >= 5. Otherwise, the Fisher exact test has been used. P-value is displayed in the above table | | | | | | | | | |

### Figure 6.3.2 Correlation coefficient matrix - Full Analysis Set Population


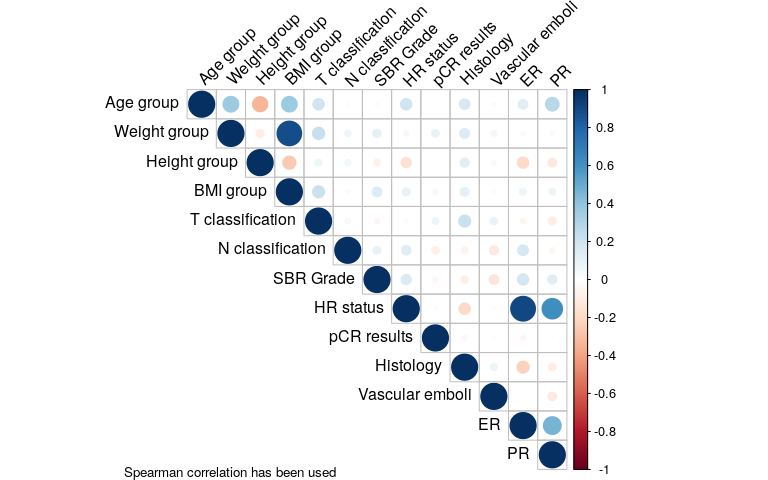

Supplement: S1 File — (DOCX) [file pone.0312697.s003.docx]
