# Supplementary material for: Capability and accuracy of usual statistical analyses in a real-world setting using a federated approach
Source: PLoS One. 2024 Nov 14;19(11):e0312697. doi: 10.1371/journal.pone.0312697 (PMC11563485; doi:10.1371/journal.pone.0312697)
Supplement: S2 File — (DOCX) [file pone.0312697.s004.docx]

PoC Federated Analytics – Analysis report on the anonymized data using DataShield

A FRENCH RETROSPECTIVE STUDY DESCRIBING THE EPIDEMIOLOGY AND THE THERAPEUTIC MANAGEMENT OF PATIENTS TREATED BY HERCEPTIN® BASED NEOADJUVANT TREATMENT FOR HER2-POSITIVE EARLY BREAST CANCER

Mathieu BOUCHER (KLS)

2023-03-23

Table of Contents

[1 Analysis of study conduct 3](#_Toc173169840)

[1.1 Patient Disposition 3](#_Toc173169841)

[Table 1.1.1 Summary of patient disposition - Full Analysis Set Population 3](#_Toc173169842)

[Table 1.1.2 Latest news - Among patients not being followed in the site - Full Analysis Set Population 4](#_Toc173169843)

[Table 1.1.3 Cause of death - Among dead patients - Full Analysis Set Population 5](#_Toc173169844)

[Table 1.1.4 Time from diagnostic to progression - Among patients having experienced progression of the disease since the beginning of adjuvant therapy - Full Analysis Set Population 6](#_Toc173169845)

[Table 1.1.5 Dates available - Full Analysis Set Population 7](#_Toc173169846)

[2 Baseline characteristics 9](#_Toc173169847)

[2.1 Demographics and baseline disease characteristics 9](#_Toc173169848)

[Table 2.1.1 Summary of demographics and baseline disease characteristics - Full Analysis Set Population 9](#_Toc173169849)

[Table 2.1.2 Summary of demographics and baseline disease characteristics by pCR result - Full Analysis Set Population 13](#_Toc173169850)

[3 Surgery and pCR 17](#_Toc173169851)

[3.1 Surgery 17](#_Toc173169852)

[3.1.1 Summary of surgery - Among patients with at least one surgery - Full Analysis Set Population 17](#_Toc173169853)

[3.2 pCR 18](#_Toc173169854)

[3.2.1 Summary of pCR - Full Analysis Set Population 18](#_Toc173169855)

[4 Adjuvant treatments 20](#_Toc173169856)

[Table 4.1 Summary of adjuvant treatments - Among subjects with at least one adjuvant treatments - Full Analysis Set Population 20](#_Toc173169857)

[Table 4.2 Summary of adjuvant treatments by adjuvant treatment - Among subjects with at least one adjuvant treatments - Full Analysis Set Population 21](#_Toc173169858)

[Table 4.3 Time between surgery and adjuvant treatment - Among subjects with at least one adjuvant treatments - Full Analysis Set Population 23](#_Toc173169859)

[Table 4.4 Summary of adjuvant treatments by pCR status - Among subjects with at least one adjuvant treatments - Full Analysis Set Population 24](#_Toc173169860)

[Table 4.5 Summary of adjuvant treatments by adjuvant treatment by pCR status - Among subjects with at least one adjuvant treatments - Full Analysis Set Population 25](#_Toc173169861)

[Table 4.6 Time between surgery and adjuvant treatment by pCR status - Among subjects with at least one adjuvant treatments - Full Analysis Set Population 27](#_Toc173169862)

[5 Efficacy Analyses 28](#_Toc173169863)

[5.1 Time to event analyses 28](#_Toc173169864)

[Table 5.1.1 Summary of time from herceptin adjuvant treatment to PFS, overall and by pCR result - Kaplan-Meier estimation - Among subjects with herceptin adjuvant treatment start date available - Full Analysis Set Population 28](#_Toc173169865)

[Table 5.1.2 Survival probabilities of time from herceptin adjuvant treatment to PFS, overall and by pCR result - Kaplan-Meier estimation - Among subjects with herceptin adjuvant treatment start date available - Full Analysis Set Population 29](#_Toc173169866)

[Table 5.1.3 Summary of time from herceptin adjuvant treatment to PFS - Kaplan-Meier curve - Among subjects with herceptin adjuvant treatment start date available - Full Analysis Set Population 30](#_Toc173169867)

[Table 5.1.4 Summary of time from herceptin adjuvant treatment to PFS by pCR result - Kaplan-Meier curve - Among subjects with herceptin adjuvant treatment start date available - Full Analysis Set Population 31](#_Toc173169868)

[6 Exploratory Analyses 32](#_Toc173169869)

[6.1 Predictive factors for PFS 32](#_Toc173169870)

[Table 6.1.1 PFS - Univariate Cox proportional hazard analysis - Among subjects with herceptin adjuvant treatment start date available - Full Analysis Set Population 32](#_Toc173169871)

[Table 6.1.2 PFS - Multivariate Cox proportional hazard analysis - Among subjects with herceptin adjuvant treatment start date available - Full Analysis Set Population 35](#_Toc173169872)

[6.2 Predictive factors for pCR result 37](#_Toc173169873)

[Table 6.2.1 pCR result - Univariate analysis - Full Analysis Set Population 37](#_Toc173169874)

[Table 6.2.2 pCR result - Multivariate analysis - Full Analysis Set Population 39](#_Toc173169875)

[6.3 Predictive factors for PFS and pCR result 40](#_Toc173169876)

[Table 6.3.1 Correlation matrix - Full Analysis Set Population 40](#_Toc173169877)

# 1 Analysis of study conduct

## 1.1 Patient Disposition

### Table 1.1.1 Summary of patient disposition - Full Analysis Set Population

| **Characteristic** | **ALL (N = 315)** |
| --- | --- |
| **Follow-up duration (years)** |  |
| Nobs | 226 |
| Mean (SD) | 4.55 (0.58) |
| Median (Q1;Q3) | 4.66 (4.46;4.85) |
| Missing | 89 |
| **Is the patient still being followed in the site (as of December 31, 2018)?, n/N (%)** |  |
| Yes | 251/306 (82%) |
| No | 55/306 (18%) |
| Missing | 9 |
| **Time from diagnostic to surgery (months)** |  |
| Nobs | 257 |
| Mean (SD) | 6.98 (2.2) |
| Median (Q1;Q3) | 6.63 (6.02;7.18) |
| Missing | 58 |
| **Has there been any progression of the disease since the beginning of adjuvant therapy, n/N (%)** |  |
| Yes | 39/304 (12.8%) |
| No | 265/304 (87.2%) |
| Missing | 11 |
| Follow-up duration (years) = (Last consultation date/Death date – initial diagnosis date of breast cancer + 1) / 365.25Time from diagnostic to surgery (months) = (Surgery date – initial diagnosis date of breast cancer) / (365.25/12) | |

### Table 1.1.2 Latest news - Among patients not being followed in the site - Full Analysis Set Population

| **Characteristic** | **ALL (N = 55)** |
| --- | --- |
| **Latest news**** |  |
| The patient is dead | 15/52 (28.8%) |
| The patient is lost to follow-up | 35/52 (67.3%) |
| Other reason | 2/52 (3.8%) |
| Missing | 3 |
| **Warning: information could be too disclosive | |

### Table 1.1.3 Cause of death - Among dead patients - Full Analysis Set Population

| **Characteristic** | **ALL (N = 15)** |
| --- | --- |
| **Cause of death**** |  |
| Disease progression | 11/13(84.6%) |
| Other reason | 2/13(15.4%) |
| Missing | 2 |
| **Warning: information could be too disclosive | |

### Table 1.1.4 Time from diagnostic to progression - Among patients having experienced progression of the disease since the beginning of adjuvant therapy - Full Analysis Set Population

| **Characteristic** | **ALL (N = 39)** |
| --- | --- |
| **Time from diagnostic to progression (years)** |  |
| Nobs | 26 |
| Mean (SD) | 2.43 (1.01) |
| Median (Q1;Q3) | 2.04 (1.85;2.97) |
| Missing | 13 |
| Time from diagnostic to progression (years) = (Date of the first progression of the disease – initial diagnosis date of breast cancer) / 365.25 | |

### Table 1.1.5 Dates available - Full Analysis Set Population

| **Characteristic** | **ALL (N = 315)** |  |
| --- | --- | --- |
| **Birth date available** |  |  |
| Yes | 315/315 (100%) |  |
| Missing | 0 |  |
| **Initial diagnosis date of breast cancer available** |  |  |
| Yes | 259/315 (82.2%) |  |
| No | 56/315 (17.8%) |  |
| Missing | 0 |  |
| **Surgery date available**** |  |  |
| Yes | 304/315 (96.5%) |  |
| No | 11/315 (3.5%) |  |
| Missing | 0 |  |
| **Date of last consultation available among patients still being followed in the site (as of December 31, 2018)** |  |  |
| Yes | 251/251 (100%) |  |
| Missing | 0 |  |
| **Date of the first progression of the disease available among patients having experienced progression of the disease since the beginning of adjuvant therapy**** |  |  |
| Yes | 34/39 (87.2%) |  |
| No | 5/39 (12.8%) |  |
| Missing | 0 |  |
| **Death date available among dead patients**** |  |  |
| Yes | 14/15(93.3%) |  |
| No | 1/15(6.7%) |  |
| Missing | 0 |  |
| **Warning: information could be too disclosive | | |

# 2 Baseline characteristics

## 2.1 Demographics and baseline disease characteristics

### Table 2.1.1 Summary of demographics and baseline disease characteristics - Full Analysis Set Population

| **Characteristic** | **ALL (N = 315)** |  |
| --- | --- | --- |
| **Age at adjuvant treatment initiation of Herceptin (years)** |  |  |
| Nobs | 303 |  |
| Mean (SD) | 52.23 (11.82) |  |
| Median (Q1;Q3) | 52.1 (43.34;60.69) |  |
| Missing | 12 |  |
| **Age group (years), n/N (%)** |  |  |
| <40 | 53/303 (17.5%) |  |
| [40 - 49] | 75/303 (24.8%) |  |
| [50 - 59] | 91/303 (30%) |  |
| [60 - 69] | 60/303 (19.8%) |  |
| >=70 | 24/303 (7.9%) |  |
| Missing | 12 |  |
| **BMI (kg/m2), n/N (%)** |  |  |
| <25 | 146/313 (46.6%) |  |
| [25 - 30[ | 117/313 (37.4%) |  |
| >=30 | 50/313 (16%) |  |
| Missing | 2 |  |
| **Professional situation, n/N (%)** |  |  |
| Worker | 162/274 (59.1%) |  |
| Jobless person | 68/274 (24.8%) |  |
| Data not found | 44/274 (16.1%) |  |
| Missing | 41 |  |
| **Weight (kg)** |  |  |
| Nobs | 314 |  |
| Mean (SD) | 67.88 (11.49) |  |
| Median (Q1;Q3) | 66 (60;73.59) |  |
| Missing | 1 |  |
| **Height (cm)** |  |  |
| Nobs | 313 |  |
| Mean (SD) | 162.99 (4.27) |  |
| Median (Q1;Q3) | 163.11 (160.5;165.33) |  |
| Missing | 2 |  |
| **Weight at initiation of adjuvant therapy (kg)** |  |  |
| Nobs | 265 |  |
| Mean (SD) | 67.34 (10.87) |  |
| Median (Q1;Q3) | 65.69 (60.55;72.41) |  |
| Missing | 50 |  |
| **Classification T, n/N (%)**** |  |  |
| T1c | 8/314 (2.5%) |  |
| T2 | 192/314 (61.1%) |  |
| T3 | 76/314 (24.2%) |  |
| T4a | 12/314 (3.8%) |  |
| T4b | 4/314 (1.3%) |  |
| T4c | 3/314 (1%) |  |
| T4d | 18/314 (5.7%) |  |
| TX | 1/314 (0.3%) |  |
| Missing | 1 |  |
| **Classification N, n/N (%)**** |  |  |
| N0 | 112/309 (36.2%) |  |
| N1 | 147/309 (47.6%) |  |
| N2 | 22/309 (7.1%) |  |
| N3 | 3/309 (1%) |  |
| NX | 25/309 (8.1%) |  |
| Missing | 6 |  |
| **Histology at the initial diagnosis, n/N (%)**** |  |  |
| Invasive ductual carcinoma | 291/306 (95.1%) |  |
| Invasive lobular carcinoma | 9/306 (2.9%) |  |
| Other | 6/306 (2%) |  |
| Missing | 9 |  |
| **Presence of vascular emboli, n/N (%)** |  |  |
| Yes | 15/230 (6.5%) |  |
| No | 215/230 (93.5%) |  |
| Missing | 85 |  |
| **SBR grade, n/N (%)**** |  |  |
| SBR I | 5/304 (1.6%) |  |
| SBR II | 139/304 (45.7%) |  |
| SBR III | 154/304 (50.7%) |  |
| Ungradable | 6/304 (2%) |  |
| Missing | 11 |  |
| **Number of lymph nodes invaded** |  |  |
| Nobs | 221 |  |
| Mean (SD) | 0.86 (1.11) |  |
| Median (Q1;Q3) | 1 (0;1) |  |
| Missing | 94 |  |
| **Estrogen receptors, n/N (%)** |  |  |
| Positive | 167/306 (54.6%) |  |
| Negative | 139/306 (45.4%) |  |
| Missing | 9 |  |
| **Progesterone receptors, n/N (%)** |  |  |
| Positive | 107/305 (35.1%) |  |
| Negative | 198/305 (64.9%) |  |
| Missing | 10 |  |
| **Warning: information could be too disclosive | | |

### Table 2.1.2 Summary of demographics and baseline disease characteristics by pCR result - Full Analysis Set Population

| **Characteristic** | **pCR (N=127)** | **No pCR (N=188)** |
| --- | --- | --- |
| **Age at adjuvant treatment initiation of Herceptin (years)** |  |  |
| Nobs | 123 | 180 |
| Mean (SD) | 52.23 (12.29) | 52.23 (11.58) |
| Median (Q1;Q3) | 51.4 (44;61) | 53.6 (43.4;60.7) |
| Missing | 4 | 8 |
| **Age group (years), n/N (%)**** |  |  |
| <40 | 23/123 (18.7%) | 30/180 (16.7%) |
| [40 - 49[ | 30/123 (24.4%) | 45/180 (25%) |
| [50 - 59[ | 36/123 (29.3%) | 55/180 (30.6%) |
| [60 - 69[ | 21/123 (17.1%) | 39/180 (21.7%) |
| >=70 | 13/123 (10.6%) | 11/180 (6.1%) |
| Missing | 4 | 8 |
| **BMI (kg/m2), n/N (%)** |  |  |
| <25 | 63/127 (49.6%) | 83/186 (44.6%) |
| [25 - 30[ | 50/127 (39.4%) | 67/186 (36%) |
| >=30 | 14/127 (11%) | 36/186 (19.4%) |
| Missing | 0 | 2 |
| **Professional situation, n/N (%)** |  |  |
| Data not found | 14/111 (12.6%) | 30/163 (18.4%) |
| Worker | 72/111 (64.9%) | 90/163 (55.2%) |
| Jobless person | 25/111 (22.5%) | 43/163 (26.4%) |
| Missing | 16 | 25 |
| **Weight (kg)** |  |  |
| Nobs | 127 | 187 |
| Mean (SD) | 66.38 (9.98) | 68.89 (12.28) |
| Median (Q1;Q3) | 64.9 (58.9;71.5) | 66.4 (61.7;75.4) |
| Missing | 0 | 1 |
| **Height (cm)** |  |  |
| Nobs | 127 | 186 |
| Mean (SD) | 163 (3.99) | 162.99 (4.38) |
| Median (Q1;Q3) | 163 (160.6;165.7) | 163 (159.8;165.3) |
| Missing | 0 | 2 |
| **Weight at initiation of adjuvant therapy (kg)** |  |  |
| Nobs | 108 | 157 |
| Mean (SD) | 66.81 (9.65) | 67.7 (11.68) |
| Median (Q1;Q3) | 65.3 (60.2;71.6) | 65.6 (60.7;73.2) |
| Missing | 19 | 31 |
| **Classification T, n/N (%)**** |  |  |
| T1c | 4/126 (3.2%) | 4/188 (2.1%) |
| T2 | 75/126 (59.5%) | 117/188 (62.2%) |
| T3 | 33/126 (26.2%) | 43/188 (22.9%) |
| T4a | 8/126 (6.3%) | 4/188 (2.1%) |
| T4b | 2/126 (1.6%) | 2/188 (1.1%) |
| T4c | 0/126 (0%) | 3/188 (1.6%) |
| T4d | 3/126 (2.4%) | 15/188 (8%) |
| TX | 1/126 (0.8%) | 0/188 (0%) |
| Missing | 1 | 0 |
| **Classification N, n/N (%)**** |  |  |
| N0 | 42/122 (34.4%) | 70/187 (37.4%) |
| N1 | 58/122 (47.5%) | 89/187 (47.6%) |
| N2 | 12/122 (9.8%) | 10/187 (5.3%) |
| N3 | 2/122 (1.6%) | 1/187 (0.5%) |
| NX | 8/122 (6.6%) | 17/187 (9.1%) |
| Missing | 5 | 1 |
| **Histology at the initial diagnosis, n/N (%)**** |  |  |
| Invasive ductual carcinoma | 116/124 (93.5%) | 175/182 (96.2%) |
| Invasive lobular carcinoma | 4/124 (3.2%) | 5/182 (2.7%) |
| Other | 4/124 (3.2%) | 2/182 (1.1%) |
| Missing | 3 | 6 |
| **Presence of vascular emboli, n/N (%)**** |  |  |
| Yes | 7/100 (7%) | 8/130 (6.2%) |
| No | 93/100 (93%) | 122/130 (93.8%) |
| Missing | 27 | 58 |
| **SBR grade, n/N (%)**** |  |  |
| SBR I | 2/122 (1.6%) | 3/182 (1.6%) |
| SBR II | 56/122 (45.9%) | 83/182 (45.6%) |
| SBR III | 63/122 (51.6%) | 91/182 (50%) |
| Ungradable | 1/122 (0.8%) | 5/182 (2.7%) |
| Missing | 5 | 6 |
| **Number of lymph nodes invaded** |  |  |
| Nobs | 94 | 127 |
| Mean (SD) | 0.73 (0.86) | 0.95 (1.25) |
| Median (Q1;Q3) | 0.7 (0;1) | 1 (0;1) |
| Missing | 33 | 61 |
| **Estrogen receptors, n/N (%)** |  |  |
| Positive | 61/124 (49.2%) | 106/182 (58.2%) |
| Negative | 63/124 (50.8%) | 76/182 (41.8%) |
| Missing | 3 | 6 |
| **Progesterone receptors, n/N (%)** |  |  |
| Positive | 45/123 (36.6%) | 62/182 (34.1%) |
| Negative | 78/123 (63.4%) | 120/182 (65.9%) |
| Missing | 4 | 6 |
| **Warning: information could be too disclosive | | |

# 3 Surgery and pCR

## 3.1 Surgery

### 3.1.1 Summary of surgery - Among patients with at least one surgery - Full Analysis Set Population

| **Characteristic** | **ALL (N = 315)** |
| --- | --- |
| **At least one Surgery*** |  |
| Axillary curage | 249/315 (79.05%) |
| Mastectomy | 169/315 (53.65%) |
| Conservative surgery | 146/315 (46.35%) |
| Sentinel Ganglion | 53/315 (16.83%) |
| * One patient can have reported several surgery types | |

## 3.2 pCR

### 3.2.1 Summary of pCR - Full Analysis Set Population

| **Characteristic** | **ALL (N = 315)** |
| --- | --- |
| **pCR results*** |  |
| pCR | 127/315 (40.3%) |
| No pCR | 188/315 (59.7%) |
| Missing | 0 |
| **Absence of invasive and in situ residues in the breast and in the lymph nodes** |  |
| Yes | 107/256 (41.8%) |
| No | 149/256 (58.2%) |
| Missing | 59 |
| **Absence of invasive residues in the breast and lymph nodes, regardless of the presence of ductal carcinoma in situ** |  |
| Yes | 108/256 (42.2%) |
| No | 148/256 (57.8%) |
| Missing | 59 |
| **Classification Chevallier**** |  |
| Grade 1 | 1/4 (25%) |
| Grade 2 | 1/4 (25%) |
| Grade 3 | 2/4 (50%) |
| Missing | 311 |
| **Classification Sataloff T**** |  |
| TA | 33/51 (64.7%) |
| TB | 15/51 (29.4%) |
| TC | 3/51 (5.9%) |
| Missing | 264 |
| **Classification Sataloff N**** |  |
| NA | 24/51 (47.1%) |
| NB | 23/51 (45.1%) |
| NC | 4/51 (7.8%) |
| Missing | 264 |
| **Classification RCB**** |  |
| RCB-II | 1/1 (100%) |
| Missing | 314 |
| * pCR results = pCR if ypT0/Tis ypN0 is ticked Yes OR, Grade 1 or Grade 2 are ticked for Classification Chevallier OR, TA and NA are ticked for Classification Sataloff OR, RCB0 is ticked for Classification RCB **Warning: information could be too disclosive | |

# 4 Adjuvant treatments

## Table 4.1 Summary of adjuvant treatments - Among subjects with at least one adjuvant treatments - Full Analysis Set Population

| **Characteristic** | **ALL (N = 305)** |
| --- | --- |
| **At least one Adjuvant Treatment*** |  |
| Trastuzumab (Herceptin) | 304/305 (99.67%) |
| Tamoxifene | 81/305 (26.56%) |
| Letrozole | 40/305 (13.11%) |
| Other | 30/305 (9.84%) |
| * One patient can have reported several adjuvant treatment types. Note: Adjuvant treatment taken by less than 30 subjects are regrouped in Other category. | |

## Table 4.2 Summary of adjuvant treatments by adjuvant treatment - Among subjects with at least one adjuvant treatments - Full Analysis Set Population

| **Characteristic** | **ALL** |
| --- | --- |
| **Trastuzumab (Herceptin)** |  |
| **Duration (months)** |  |
| **Nobs** | **297** |
| Mean (SD) | 8.14 (2.98) |
| Median (Q1;Q3) | 8.86 (7.84;9.22) |
| Missing | 7 |
| **Administration frequency** |  |
| Nobs | 257 |
| Mean (SD) | 3.02 (0.15) |
| Median (Q1;Q3) | 3 (3;3) |
| Missing | 47 |
| **Maintenance dose (cycle)** |  |
| Nobs | 264 |
| Mean (SD) | 7.76 (23.79) |
| Median (Q1;Q3) | 6 (6;6) |
| Missing | 40 |
| **Maintenance dose (cycle mg/kg or mg)** |  |
| Nobs | 30 |
| Mean (SD) | 572 (81.2) |
| Median (Q1;Q3) | 600 (594.25;600) |
| Missing | 274 |
| **Route of administration, n/N (%)** |  |
| Subcutaneous | 59/261 (22.6%) |
| Intravenous | 162/261 (62.1%) |
| Both | 40/261 (15.3%) |
| Missing | 43 |
| **Number of cycles completed** |  |
| Nobs | 294 |
| Mean (SD) | 13.3 (2.55) |
| Median (Q1;Q3) | 13.8 (12.15;14.5) |
| Missing | 10 |
| **Location of administration, n/N (%)** |  |
| Hospital | 277/283 (97.9%) |
| Home | 6/283 (2.1%) |
| Missing | 21 |
| **Start date of treatment available, n/N (%)** |  |
| Yes | 303/304 (99.67%) |
| No | 1/304 (0.33%) |
| **End date of treatment available, n/N (%)** |  |
| Yes | 297/304 (97.7%) |
| No | 7/304 (2.3%) |
| Duration of each adjuvant (months) = (End date of treatment – Start date of treatment + 1) / (365.25/12) | |

## Table 4.3 Time between surgery and adjuvant treatment - Among subjects with at least one adjuvant treatments - Full Analysis Set Population

| **Characteristic** | **ALL (N = 304)** |
| --- | --- |
| **Time from surgery to adjuvant treatment initiation of Herceptin (days)** |  |
| Nobs | 301 |
| Mean (SD) | 84.1 (1768.07) |
| Median (Q1;Q3) | 23.68 (-1061.8;1150.33) |
| Missing | 3 |
| Time from surgery to adjuvant treatment initiation of Herceptin (days) = (Date of adjuvant treatment initiation of Herceptin - Surgery date) | |

## Table 4.4 Summary of adjuvant treatments by pCR status - Among subjects with at least one adjuvant treatments - Full Analysis Set Population

| **Characteristic** | **pCR (N = 124)** | **No pCR (N = 181)** |
| --- | --- | --- |
| **At least one Adjuvant Treatment*** |  |  |
| Trastuzumab (Herceptin)** | 124/124 (100%) | 180/181 (99.45%) |
| Tamoxifene** | 30/124 (24.19%) | 51/181 (28.18%) |
| Letrozole** | 16/124 (12.9%) | 24/181 (13.26%) |
| * One patient can have reported several adjuvant treatment types **Warning: information could be too disclosive | | |

## Table 4.5 Summary of adjuvant treatments by adjuvant treatment by pCR status - Among subjects with at least one adjuvant treatments - Full Analysis Set Population

| **Characteristic** | **No pCR (N=180)** | **pCR (N=124)** |
| --- | --- | --- |
| **Trastuzumab (Herceptin)** |  |  |
| **Duration (months)** |  |  |
| Nobs | 175 | 122 |
| Mean (SD) | 7.94 (3.59) | 8.42 (1.73) |
| Median (Q1;Q3) | 8.8 (7.7;9.2) | 8.9 (8.1;9.3) |
| Missing | 5 | 2 |
| **Administration frequency** |  |  |
| Nobs | 157 | 100 |
| Mean (SD) | 3.01 (0.11) | 3.04 (0.2) |
| Median (Q1;Q3) | 3 (3;3) | 3 (3;3) |
| Missing | 23 | 24 |
| **Maintenance dose (cycle)** |  |  |
| Nobs | 158 | 106 |
| Mean (SD) | 8.53 (30.62) | 6.62 (3.64) |
| Median (Q1;Q3) | 6 (6;6) | 6 (6;6) |
| Missing | 22 | 18 |
| **Maintenance dose (cycle mg/kg or mg)** | **n<3 in at least one split** |  |
| **Route of administration, n/N (%)**** |  |  |
| Subcutaneous | 31/155 (20%) | 28/106 (26.4%) |
| Intravenous | 99/155 (63.9%) | 63/106 (59.4%) |
| Both | 25/155 (16.1%) | 15/106 (14.2%) |
| Missing | 25 | 18 |
| **Number of cycles completed** |  |  |
| Nobs | 173 | 121 |
| Mean (SD) | 13.32 (2.47) | 13.26 (2.7) |
| Median (Q1;Q3) | 13.9 (12;14.1) | 13.8 (12.5;14.5) |
| Missing | 7 | 3 |
| **Location of administration, n/N (%)**** |  |  |
| Hospital | 164/167 (98.2%) | 113/116 (97.4%) |
| Home | 3/167 (1.8%) | 3/116 (2.6%) |
| Missing | 13 | 8 |
| **End date of treatment available, n/N (%)**** |  |  |
| Yes | 175/175 (100%) | 122/122 (100%) |
| Missing | 5 | 2 |
| **Start date of treatment available, n/N (%)**** |  |  |
| Yes | 180/180 (100%) | 123/123 (100%) |
| Missing | 0 | 1 |
| Duration of each adjuvant (months) = (End date of treatment – Start date of treatment + 1) / (365.25/12) **Warning: information could be too disclosive | | |

## Table 4.6 Time between surgery and adjuvant treatment by pCR status - Among subjects with at least one adjuvant treatments - Full Analysis Set Population

| **Characteristic** | **No pCR (N=180)** | **pCR (N=124)** |
| --- | --- | --- |
| **Time from surgery to adjuvant treatment initiation of Herceptin (days)** |  |  |
| Nobs | 180 | 121 |
| Mean (SD) | 163.16 (1815.17) | -33.5 (1688.6) |
| Median (Q1;Q3) | 156.9 (-1110.9;1311.9) | -93 (-1015.4;896) |
| Missing | 0 | 3 |
| Time from surgery to adjuvant treatment initiation of Herceptin (days) = (Date of adjuvant treatment initiation of Herceptin - Surgery date) | | |

# 5 Efficacy Analyses

## 5.1 Time to event analyses

### Table 5.1.1 Summary of time from herceptin adjuvant treatment to PFS, overall and by pCR result - Kaplan-Meier estimation - Among subjects with herceptin adjuvant treatment start date available - Full Analysis Set Population

| **Strata** | **N** | **Number of event**** | **10% Percentile (95% CI)** |
| --- | --- | --- | --- |
| All | 297 | 37 | 6.39 (-0.27;11.21) |
| pCR results* |  |  |  |
| No pCR | 175 | 28 | 2.04 (-2.31;7.4) |
| pCR | 122 | 9 | 10.31 (1.29;0) |
| Patients who did not experience event were censored at their last consultation date. If this date is missing, they were censored at the last adjuvant treatment date. * pCR results = pCR if ypT0/Tis ypN0 is ticked Yes OR, Grade 1 or Grade 2 are ticked for Classification Chevallier OR, TA and NA are ticked for Classification Sataloff OR, RCB0 is ticked for Classification RCB. ** Warning: information could be too disclosive | | | |

### Table 5.1.2 Survival probabilities of time from herceptin adjuvant treatment to PFS, overall and by pCR result - Kaplan-Meier estimation - Among subjects with herceptin adjuvant treatment start date available - Full Analysis Set Population

Warning: following information could be too disclosive!

| **Strata** | **At 1 years (95%IC)** | **At 2 years (95%IC)** | **At 3 years (95%IC)** | **At 4 years (95%IC)** |
| --- | --- | --- | --- | --- |
| All | 93.5 (88.8;99) | 92.6 (87.5;98) | 92.6 (87.5;98) | 90.6 (84.7;97) |
| pCR results* |  |  |  |  |
| pCR | 96.6 (92.3;100) | 95.5 (90.6;100) | 95.5 (90.6;100) | 94.1 (88.2;100) |
| No pCR | 91.4 (84.4;99) | 90.5 (83.1;98) | 90.5 (83.1;98) | 88.4 (79.8;98) |
| Patients who did not experience event were censored at their last consultation date. If this date is missing, they were censored at the last adjuvant treatment date. * pCR results = pCR if ypT0/Tis ypN0 is ticked Yes OR, Grade 1 or Grade 2 are ticked for Classification Chevallier OR, TA and NA are ticked for Classification Sataloff OR, RCB0 is ticked for Classification RCB | | | | |

### Table 5.1.3 Summary of time from herceptin adjuvant treatment to PFS - Kaplan-Meier curve - Among subjects with herceptin adjuvant treatment start date available - Full Analysis Set Population

Warning: following information could be too disclosive!


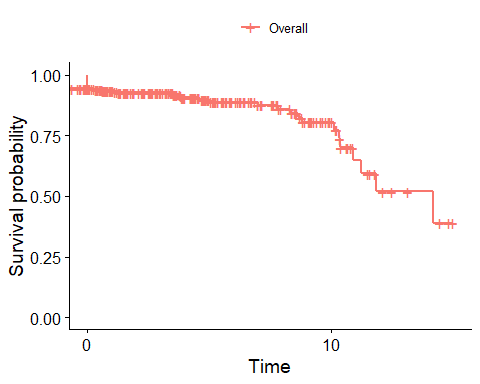


### Table 5.1.4 Summary of time from herceptin adjuvant treatment to PFS by pCR result - Kaplan-Meier curve - Among subjects with herceptin adjuvant treatment start date available - Full Analysis Set Population

Warning: following information could be too disclosive!


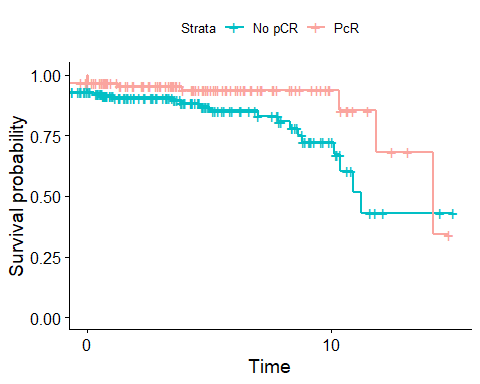


# 6 Exploratory Analyses

## 6.1 Predictive factors for PFS

### Table 6.1.1 PFS - Univariate Cox proportional hazard analysis - Among subjects with herceptin adjuvant treatment start date available - Full Analysis Set Population

| **Characteristic** | **No event (N=260)** | **Event (N=43)** | **HR** | **CI** | **pval** |
| --- | --- | --- | --- | --- | --- |
| **Age at adjuvant treatment initiation of Herceptin (years)** |  |  | **0.99** | **[0.96;1.03]** | **0.2662** |
| Nobs | 260 | 43 |  |  |  |
| Mean (SD) | 52.49 (11.63) | 50.65 (12.65) |  |  |  |
| Median (Q1;Q3) | 52.4 (43.6;60.7) | 52.5 (42.8;58.4) |  |  |  |
| Missing | 0 | 0 |  |  |  |
| **Age group (years)**** |  |  |  |  | **0.5415** |
| <40 | 41/260 (15.8%) | 12/43 (27.9%) |  |  |  |
| [40 - 49[ | 70/260 (26.9%) | 5/43 (11.6%) | 0.28 | [0.09;0.86] |  |
| [50 - 59[ | 75/260 (28.8%) | 16/43 (37.2%) | 1.27 | [0.55;2.93] |  |
| [60 - 69[ | 52/260 (20%) | 8/43 (18.6%) | 0.68 | [0.22;2.07] |  |
| >=70 | 22/260 (8.5%) | 2/43 (4.7%) | 0.86 | [0.18;4.17] |  |
| Missing | 0 | 0 |  |  |  |
| **BMI (kg/m2)**** |  |  |  |  | **0.7282** |
| <25 | 122/259 (47.1%) | 19/42 (45.2%) |  |  |  |
| [25 - 30[ | 96/259 (37.1%) | 16/42 (38.1%) | 0.79 | [0.27;2.32] |  |
| >=30 | 41/259 (15.8%) | 7/42 (16.7%) | 1.50 | [0.57;3.97] |  |
| Missing | 1 | 1 |  |  |  |
| **pCR results**** |  |  |  |  | **0.3418** |
| No pCR | 147/260 (56.5%) | 33/43 (76.7%) | 2.06 | [0.75;5.67] |  |
| pCR | 113/260 (43.5%) | 10/43 (23.3%) |  |  |  |
| Missing | 0 | 0 |  |  |  |
| **Presence of vascular emboli**** |  |  |  |  | **0.0123** |
| Yes | 10/199 (5%) | 5/29 (17.2%) |  |  |  |
| No | 189/199 (95%) | 24/29 (82.8%) | 0.23 | [0.06;0.9] |  |
| Missing | 61 | 14 |  |  |  |
| **Hormonal receptors status**** |  |  |  |  | **0.2709** |
| ER and/or PR + | 158/259 (61%) | 23/43 (53.5%) |  |  |  |
| ER and PR - | 101/259 (39%) | 20/43 (46.5%) | 1.87 | [0.95;3.69] |  |
| Missing | 1 | 0 |  |  |  |
| **SBR Grade**** |  |  |  |  | **0.6196** |
| SBR I & II | 125/258 (48.4%) | 18/43 (41.9%) |  |  |  |
| SBR III | 128/258 (49.6%) | 24/43 (55.8%) | 1.51 | [0.78;2.93] |  |
| Missing | 7 | 1 |  |  |  |
| **T classification**** |  |  |  |  | **0.2245** |
| T0-3 | 236/258 (91.5%) | 31/43 (72.1%) |  |  |  |
| T>3 | 22/258 (8.5%) | 12/43 (27.9%) | 2.10 | [0.96;4.56] |  |
| Missing | 2 | 0 |  |  |  |
| **N classification**** |  |  |  |  | **0.8508** |
| N0 | 91/232 (39.2%) | 17/42 (40.5%) |  |  |  |
| N1 | 120/232 (51.7%) | 22/42 (52.4%) | 1.27 | [0.62;2.58] |  |
| N2&N3 | 21/232 (9.1%) | 3/42 (7.1%) | 1.37 | [0.28;6.69] |  |
| Missing | 28 | 1 |  |  |  |
| Univariate analysis has been done using a cox model. P-values are combined using Fisher's method. HR = Hazard Ratio, CI = Confidence Interval. **Warning: information could be too disclosive. | | | | | |

### Table 6.1.2 PFS - Multivariate Cox proportional hazard analysis - Among subjects with herceptin adjuvant treatment start date available - Full Analysis Set Population

| **Characteristic** | **No event (N=260)** | **Event (N=43)** | **HR** | **CI** | **pval** |
| --- | --- | --- | --- | --- | --- |
| **Presence of vascular emboli**** |  |  |  |  | **0.0470** |
| Yes | 10/199 (5%) | 5/29 (17.2%) |  |  |  |
| No | 189/199 (95%) | 24/29 (82.8%) | 0.12 | [0.04;0.37] |  |
| Missing | 61 | 14 |  |  |  |
| **Age group (years)**** |  |  |  |  | **0.0377** |
| <40 | 41/260 (15.8%) | 12/43 (27.9%) |  |  |  |
| [40 - 49[ | 70/260 (26.9%) | 5/43 (11.6%) | 0.00 | [0;Inf] |  |
| [50 - 59[ | 75/260 (28.8%) | 16/43 (37.2%) | 0.00 | [0;823.78] |  |
| [60 - 69[ | 52/260 (20%) | 8/43 (18.6%) | 0.34 | [0.09;1.34] |  |
| >=70 | 22/260 (8.5%) | 2/43 (4.7%) | 0.62 | [0.11;3.47] |  |
| Missing | 0 | 0 |  |  |  |
| **pCR results**** |  |  |  |  | **0.0620** |
| No pCR | 147/260 (56.5%) | 33/43 (76.7%) |  |  |  |
| pCR | 113/260 (43.5%) | 10/43 (23.3%) | 3.66 | [1.15;11.66] |  |
| Missing | 0 | 0 |  |  |  |
| **T classification**** |  |  |  |  | **0.0451** |
| T0-3 | 236/258 (91.5%) | 31/43 (72.1%) |  |  |  |
| T>3 | 22/258 (8.5%) | 12/43 (27.9%) | 2,681.54 | [0;2790480047.55] |  |
| Missing | 2 | 0 |  |  |  |
| Multivariate analysis has been done using a cox model. For covariate with 2 modalities, p-value is based on a global wald test from Cox model, otherwise it is the likelihood ratio test global p-value. The model has been constructed using a stepwise selection of covariates with 0.15 as entry threshold and 0.15 as the retention threshold. P-values are combined using Fisher's method. HR = Hazard Ratio, CI = Confidence Interval. **Warning: information could be too disclosive. | | | | | |

## 6.2 Predictive factors for pCR result

### Table 6.2.1 pCR result - Univariate analysis - Full Analysis Set Population

| **Characteristic** | **No pCR (N=188)** | **pCR (N=127)** | **OR** | **95% CI** | **pval** |
| --- | --- | --- | --- | --- | --- |
| **Age at adjuvant treatment initiation of Herceptin (years)** |  |  | **1** | **[0.98;1.02]** | **0.7354** |
| Nobs | 180 | 123 |  |  |  |
| Mean (SD) | 52.23 (11.58) | 52.23 (12.29) |  |  |  |
| Median (Q1;Q3) | 53.6 (43.4;60.7) | 51.4 (44;61) |  |  |  |
| Missing | 8 | 4 |  |  |  |
| **Age group (years), n/N (%)**** |  |  |  |  | **0.9002** |
| <40 | 30/180 (16.7%) | 23/123 (18.7%) |  |  |  |
| [40 - 49[ | 45/180 (25%) | 30/123 (24.4%) | 0.86 | [0.41;1.77] |  |
| [50 - 59[ | 55/180 (30.6%) | 36/123 (29.3%) | 0.86 | [0.43;1.72] |  |
| [60 - 69[ | 39/180 (21.7%) | 21/123 (17.1%) | 0.74 | [0.34;1.6] |  |
| >=70 | 11/180 (6.1%) | 13/123 (10.6%) | 1.54 | [0.57;4.13] |  |
| Missing | 8 | 4 |  |  |  |
| **BMI (kg/m2), n/N (%)** |  |  |  |  | **0.1181** |
| <25 | 83/186 (44.6%) | 63/127 (49.6%) |  |  |  |
| [25 - 30[ | 67/186 (36%) | 50/127 (39.4%) | 0.98 | [0.6;1.61] |  |
| >=30 | 36/186 (19.4%) | 14/127 (11%) | 0.56 | [0.15;2.03] |  |
| Missing | 2 | 0 |  |  |  |
| **Classification T, n/N (%)**** |  |  |  |  | **0.4048** |
| T0-3 | 164/188 (87.2%) | 112/125 (89.6%) |  |  |  |
| T>3 | 24/188 (12.8%) | 13/125 (10.4%) | 0.82 | [0.37;1.83] |  |
| Missing | 0 | 2 |  |  |  |
| **N classification, n/N (%)**** |  |  |  |  | **0.1188** |
| N0 | 70/170 (41.2%) | 42/114 (36.8%) |  |  |  |
| N1 | 89/170 (52.4%) | 58/114 (50.9%) | 0.86 | [0.31;2.36] |  |
| N2&N3 | 11/170 (6.5%) | 14/114 (12.3%) | 2.06 | [0.85;4.96] |  |
| Missing | 18 | 13 |  |  |  |
| **SBR grade, n/N (%)**** |  |  |  |  | **0.2203** |
| SBR I & II | 86/177 (48.6%) | 58/121 (47.9%) |  |  |  |
| SBR III | 91/177 (51.4%) | 63/121 (52.1%) | 0.94 | [0.48;1.88] |  |
| Missing | 11 | 6 |  |  |  |
| **Presence of vascular emboli, n/N (%)**** |  |  |  |  | **0.9931** |
| Yes | 8/130 (6.2%) | 7/100 (7%) |  |  |  |
| No | 122/130 (93.8%) | 93/100 (93%) | 0.88 | [0.3;2.53] |  |
| Missing | 58 | 27 |  |  |  |
| **Hormonal receptors status** |  |  |  |  | **0.4362** |
| ER and/or PR + | 111/182 (61%) | 72/123 (58.5%) |  |  |  |
| ER and PR - | 71/182 (39%) | 51/123 (41.5%) | 1.08 | [0.62;1.86] |  |
| Missing | 6 | 4 |  |  |  |
| Univariate analysis has been done using a logistic model. P-value are associated to 95%CI of each modality.OR = Odds Ratio, CI = Confidence Interval | | | | | |

### Table 6.2.2 pCR result - Multivariate analysis - Full Analysis Set Population

No multivariate model available at 0.15 level.

## 6.3 Predictive factors for PFS and pCR result

### Table 6.3.1 Correlation matrix - Full Analysis Set Population

| Variables | Age (years) | Age group (years) | BMI (kg/m2) | T classification | N classification | SBR Grade | Presence of vascular emboli | Hormonal receptors status | pCR results |
| --- | --- | --- | --- | --- | --- | --- | --- | --- | --- |
| Age (years) | ND |  |  |  |  |  |  |  |  |
| Age group (years) | ND | ND |  |  |  |  |  |  |  |
| BMI (kg/m2) | 0 | 5e-04 | ND |  |  |  |  |  |  |
| T classification | 0.2679 | 0.026 | 0.0142 | ND |  |  |  |  |  |
| N classification | 9e-04 | 0.022 | 0.4921 | 0.0456 | ND |  |  |  |  |
| SBR Grade | 0.8403 | 0.9471 | 0.0356 | 1 | 0.0819 | ND |  |  |  |
| Presence of vascular emboli | 0.3867 | 0.9045 | 0.8036 | 0.3723 | 0.1664 | 0.285 | ND |  |  |
| Hormonal receptors status | 0.0059 | 0.0413 | 0.092 | 0.1132 | 0.0021 | 0.011 | 0.6001 | ND |  |
| pCR results | 0.7409 | 0.5857 | 0.1418 | 0.6482 | 0.2264 | 1 | 1 | 0.7568 | ND |
| * pCR results = pCR if ypT0/Tis ypN0 is ticked Yes OR, Grade 1 or Grade 2 are ticked for Classification Chevallier OR, TA and NA are ticked for Classification Sataloff OR, RCB0 is ticked for Classification RCB | | | | | | | | | |
| ND: Not Done | | | | | | | | | |
| Between quantitative and qualitative variables: Anova have been used: the p-value displayed is the p-value of the Type 3 test of fixed effects. P-value is displayed in the above table. | | | | | | | | | |
| Between qualitative variables: Chi² test has been used when all expected counts are >= 5. Otherwise, the Fisher exact test has been used. P-value is displayed in the above table | | | | | | | | | |
